# Introduced plants as novel Anthropocene habitats for insects

**DOI:** 10.1111/gcb.14915

**Published:** 2019-12-16

**Authors:** Roberto J. Padovani, Andrew Salisbury, Helen Bostock, David B. Roy, Chris D. Thomas

**Affiliations:** ^1^ Leverhulme Centre for Anthropocene Biodiversity University of York York UK; ^2^ Royal Horticultural Society Surrey UK; ^3^ Centre for Ecology & Hydrology Wallingford UK

**Keywords:** biodiversity, conservation biology, entomology, environmental change, macroecology, phylogenetics, phytophagous

## Abstract

Major environmental changes in the history of life on Earth have given rise to novel habitats, which gradually accumulate species. Human‐induced change is no exception, yet the rules governing species accumulation in anthropogenic habitats are not fully developed. Here we propose that nonnative plants introduced to Great Britain may function as analogues of novel anthropogenic habitats for insects and mites, analysing a combination of local‐scale experimental plot data and geographic‐scale data contained within the Great Britain Database of Insects and their Food Plants. We find that novel plant habitats accumulate the greatest diversity of insect taxa when they are widespread and show some resemblance to plant habitats which have been present historically (based on the relatedness between native and nonnative plant species), with insect generalists colonizing from a wider range of sources. Despite reduced per‐plant diversity, nonnative plants can support distinctive insect communities, sometimes including insect taxa that are otherwise rare or absent. Thus, novel plant habitats may contribute to, and potentially maintain, broader‐scale (assemblage) diversity in regions that contain mixtures of long‐standing and novel plant habitats.

## Graphical abstract

Ten per cent of insect species in a geographic‐scale insect–plant database (DBIF) are unique to nonnative plants (a—red text), and a mixed landscape composed of 1/3 neophyte plants (nonnatives arrived since 1500), 1/3 archaeophytes (nonnatives arrived before 1500) and 1/3 natives accumulates insect species at a similar rate to a native landscape (b). These results suggest that despite reduced per‐plant diversity, nonnative plants support distinctive insect communities, including taxa that are otherwise rare or absent on native plants. Thus, novel plant habitats contribute to broader‐scale (assemblage) diversity in regions that contain mixtures of long‐standing and novel plant habitats.

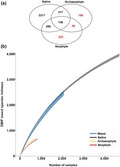

## 1 INTRODUCTION

New physical environments and unprecedented mixtures of species arriving from different geographic origins are progressively generating novel ecosystems (Evers et al., 2018; Hobbs et al., 2006; Radeloff et al., 2015) during the Anthropocene, the proposed geological epoch of humanity. The establishment of species in new abiotic and biotic environments may explain why biodiversity is typically increasing at a regional scale, whilst often relatively stable locally (with both gains and losses) and declining globally (Dornelas et al., 2014, 2019; Loh et al., 2005; Sax & Gaines, 2003; Thomas, 2013a, 2013b, 2015; Vellend et al., 2013, 2017). Thus, it is important to understand the ecological and evolutionary ‘rules’ that govern the accumulation of species in novel situations, just as it is important to identify the processes that result in extinction. Studies of brown field sites, mine tailings, old fields and green rooves have investigated the extent to which novel habitat biotas differ from those of pre‐existing habitats (Hobbs et al., 2006; Jones & Leather, 2012; Tischew, Baasch, Grunert, & Kirmer, 2014), and have demonstrated that the age of a novel habitat can influence species richness, abundance, specialism and community composition (Cramer, Hobbs, & Standish, 2008; Li, Wen, Chen, & Yin, 2014; Nichols & Nichols, 2003). However, research is constrained by the potentially unique nature of each novel habitat, and hence there is difficulty generalising about the differences seen in the accumulation of species in different types of novel habitats.

We suggest a new model system in order to achieve replication among habitat types: the association of ‘insect’ (including some mites) faunas with introduced plants in two datasets (from local‐scale experiments and a geographic‐scale database), where each introduced plant species is hypothesized to represent a different novel habitat for insects, in the regions to which the plants have been introduced. The degree to which a plant species captures all aspects of habitat may vary across trophic levels and with insect specialism (i.e. higher trophic levels may be less closely associated with individual plant species, likewise for generalists at all trophic levels—Bezemer, Harvey, & Cronin, 2014; Harvey, Bukovinszky, & van der Putten, 2010), and there are aspects of habitat that cannot be determined by plant species identity (i.e. the composition of surrounding plant communities, and site‐specific biotic and abiotic conditions). However, we suggest that plant species identity may effectively capture many aspects of habitat, with plants typically presenting specific abiotic and biotic conditions, such as varying microclimate (e.g. moisture, temperate and light intensity), chemical composition, architecture (e.g. size, branching complexity, surface and interior composition), phenology and associated faunal and microbial communities (Schoonhoven, van Loon, & Dicke, 2005; Strong, Lawton, & Southwood, 1984).

Nonnative plants are increasingly prominent in landscapes around the world (van Kleunen et al., 2015), and are now recognized to have potential conservation value due to their provision of multiple ecosystem services (Schlaepfer, Sax, & Olden, 2011), such as the hosting of complex, multitrophic, insect communities (Harvey et al., 2010). Previous comparisons of insects (mainly herbivores) associated with native and nonnative plants have generated conflicting results, in which herbivore species richness, abundance, biomass and damage to plant tissues can be lower, similar or even higher on the nonnative plants (Agrawal et al., 2005; Ando, Utsumi, & Ohgushi, 2010; Brändle, Kühn, Klotz, Belle, & Brandl, 2008; Carpenter & Cappuccino, 2005; Dostál et al., 2013; Harvey, Nipperess, Britton, & Hughes, 2013; Hawkes, 2007; Novotny et al., 2003; Strong et al., 1984; Sugiura, Yamaura, & Makihara, 2008). Some of this variation can potentially be explained by the fact that nonnative plants differ in the extent to which they are distinct from native plants. This distinctiveness emerges from differing host plant phenotypes, with these differences determined in part by the phylogenetic relationship between introduced plants and native plant species, which influences how similar they are across an array of phenotypic traits that affect how insects associate with a plant (e.g. olfactory attractants, toxic secondary plant compounds, phenology and physical structure—Bezemer et al., 2014; Cappuccino & Arnason, 2006; Rasmann & Agrawal, 2011; Schoonhoven et al., 2005). This has primarily been considered in relation to whether an introduced plant is a close relative of (e.g. in the same genus as) a native species (Branco, Brockerhoff, Castagneyrol, Orazio, & Jactel, 2015; Burghardt & Tallamy, 2015; Kirichenko & Kenis, 2016; Salisbury et al., 2015, 2017), but phylogenetic distinctiveness (our proxy for the novelty of a new plant habitat) is more complex than a binary congeneric/noncongeneric classification. When quantified as a phylogenetic isolation (Mya), phylogenetic distinctiveness can affect the diversity of insects on both native (Vialatte et al., 2010) and introduced plants (Grandez‐Rios, Bergamini, Santos de Araújo, Villalobos, & Almeida‐Neto, 2015) by influencing their phenotype. Thus, phylogenetic isolation is a convenient proxy for the degree of novelty.

In addition to the ‘degree of novelty’ of a novel habitat, it is also important to consider its age (hypothesized as the length of time that a nonnative plant has existed in a region), and its geographic extent (analogous to the range size of a nonnative plant). As the range size of an introduced plant increases over time, more potential colonists are likely to encounter it and develop specialized adaptations, potentially generating a positive correlation between the time since arrival of nonnative plant species and insect herbivore species richness (Brändle et al., 2008; Kennedy & Southwood, 1984; Kirichenko & Kenis, 2016). However, the effect of time is not always apparent (Andow & Imura, 1994; Carpenter & Cappuccino, 2005) and may be overshadowed by the effect of range size once a time‐richness asymptote is approached (Banerjee, 1981; Kennedy & Southwood, 1984; Strong, 1974; Strong, McCoy, & Rey, 1977). Host plant range size has a well‐established influence on the species richness of insects found on both native and nonnative plants (Andow & Imura, 1994; Branco et al., 2015; Brändle & Brandl, 2001; Kennedy & Southwood, 1984; Lawton, Lewinsohn, & Compton, 1993; Strong et al., 1977). However, there may be differences in the strength of the effect between natives and nonnatives (Brändle et al., 2008), and it is very rarely considered in tandem with host plant habitat novelty, that is, in relation to host plant phenotype as influenced by the phylogenetic isolation of nonnative plants (Branco et al., 2015).

Here we hypothesize that the extensive insect fauna associated with introduced plants may function as a model system for the accumulation of species in novel anthropogenic habitats. We consider several functional groups and trophic levels (herbivores, detritivores, omnivores, predators and pollinators), because the ‘perception of novelty’ by colonizing insects may vary among functional groups and trophic levels (Ando et al., 2010; Fortuna et al., 2013; Salisbury et al., 2017). We test whether (a) novel plant habitats accumulate the greatest diversity of associated taxa when they show some resemblance to surrounding habitats which have been present historically, and (b) the recruitment of taxa into novel plant habitats varies among functional/trophic groupings. We also (c) test whether phylogenetically distinct plants accumulate divergent biological assemblages, and hence (d) whether this divergence may retain or increase diversity in areas that contain mixtures of long‐standing and novel plant habitats. Finally, we test the hypotheses that (e) novel plant habitat age (time since introduction of nonnative plants which have been introduced since 1500) and (f) geographic extent (host plant range size) influence the accumulation of diversity and the composition of biological assemblages. This paper tests these hypotheses at two spatial scales using complementary data sets: an extensive field experiment spanning several years (2010–2016), which examines the insects sampled from 69 garden plant species that vary in their relatedness to the native flora of Great Britain; and analysis of the insect–plant interactions contained within the Great Britain Database of Insects and their Food Plants (DBIF).

## 2 MATERIALS AND METHODS

### 2.1 Local‐scale: The experimental plots

The experimental plots were located on two 25 × 13 m sites (blocks) at Wisley, Surrey, UK; one located within the Royal Horticultural Society's Wisley Garden at Howard's Field, and the other at the adjacent Deers Farm. Each site housed eighteen 3 × 3 m plots, and each plot contained 14 plant species, drawn from a total list of 69 plant species typically found in flower gardens in Great Britain. The 69 plant species were organized into 23 species triplets, with a third of the plots containing a mixture of native plant species, a third containing a mixture of nonnative species closely related to the natives (‘congeners’), and the remaining third of the plots containing a mixture of distantly or unrelated ‘exotic’ plant species from the southern hemisphere (see below). There were nine different plant mixtures in total (three of native species; three of congeners and three of exotics), with each occurring twice at each experimental site. Mixtures were assigned locations on sites using restricted randomization, ensuring an even distribution of plots along the north–south direction. Species replication (4–12 times across the two sites depending on the species triplet) allowed us to test for the effects of species ‘native status’ (native, nonnative congener, exotic) and phylogenetic isolation. The Supporting Information provides further details on the plot design and maintenance, and Table S1 includes a full list of plant species.

The location of plant species on the plots followed a standardized pattern, and controlled for plant growth forms and architectures. Plant species in the same location on each plot (irrespective of native status) were chosen to be as similar as possible in terms of plant height, density and structure, ensuring that the overall composition of each plot was analogous. Initial planting took place between May 2009 and June 2010 (see Supporting Information for further details).

The three native status categories were defined geographically and taxonomically:

1. *Native*: A species that arrived in Great Britain without anthropogenic intervention (Pyšek et al., 2004).
2. *Congener*: A species occurring naturally only in the Northern Hemisphere, but not native or naturalized in Great Britain. They were matched by growth habit with the corresponding native plant in the same experiments. ‘Congeners’ were usually congeneric (16/23) with this native plant, but in seven instances were confamilial. For simplicity, they are collectively referred to as ‘congeners’.
3. *Exotic*: A species occurring naturally only in the Southern Hemisphere, and not naturalized in Great Britain. They were matched in terms of growth habit with the corresponding native plant, and were not necessarily related to it at any particular taxonomic rank. In three cases exotics were confamilial with the native, but in all other cases were more distantly related.

### 2.2 Local‐scale: Sampling flower visiting aerial insects (pollinators) on experimental plots

Flower visiting aerial insects (hereafter ‘pollinators’) were sampled from 2010 to 2013, over four to five sampling days per year, with a minimum of 4 weeks between days. Sampling days occurred from March to September, covering the main period of pollinator activity, and under climatic conditions that were favourable to pollinator activity. During each sampling session an expert in insect identification (A. Salisbury) stood at the centre of each of the four sides of a plot for 1 min and counted all flying insects that landed on or were already on flowers (4 min per plot total). Pollinators were identified to species level where possible, although in some cases this was not possible (35 taxa at species level, genus = 5, family = 5, superfamily = 1, infraorder = 2, suborder = 1, order = 5). For further details of the pollinator sampling protocol, see Supporting Information.

Floral resource availability was quantified, based on the methodology of Heard et al. (2007), as the estimated number of flowering units (single flower or umbel, spike or capitulum for species with reduced or compound flowers) on each plant species (excluding grasses, ferns and analogous plants) at each sampling session. Estimates were recorded as the median value from one of the following classes: 0, 1–5, 6–20, 21–100, 101–500 and 501–1,000. Flowering units >0 was a requirement for inclusion in statistical analysis.

### 2.3 Local‐scale: Vortis sampling of insects on plants (various functional groups) on experimental plots

Plant inhabiting insects were sampled with a Vortis suction sampler (Arnold, 1994; Burkard Manufacturing Co. Limited) in July 2016. Vortis sampling occurred after 10:00 a.m., when vegetation was dry to the touch, and with temperatures greater than 17°C. The two experimental sites were sampled alternately, with sampling sessions rotating between them. Vortis sampling was carried out by sweeping the suction nozzle across half of each individual plant for 30 s. Certain plant species had an architecture that made efficient Vortis sampling very difficult (e.g. low‐growing plants that would generate soil contamination), and so 24 plant species were excluded from further processing and analysis. For further details of the Vortis suction sampling protocol, see Supporting Information.

### 2.4 Local‐scale: Vortis plant architecture on experimental plots

Several measures of plant architecture were taken, to account for the potential effects of plant size and complexity on insect species richness and abundance (Brändle & Brandl, 2001; Kennedy & Southwood, 1984; Morse, Lawton, Dodson, & Williamson, 1985). All plant architecture measurements were taken within a maximum of 8 days following Vortis sampling. The height of each plant was measured directly with a 3 m rule, from the ground to the height at which the main bulk of its canopy terminated. Plant area was measured using one of three methods, depending on the composition of the plots. See Supporting Information for further details. The branching architecture of the median height individual of each plant species was also measured, in order to quantify each species’ architectural complexity (Pérez‐Harguindeguy et al., 2013). See Supporting Information for further details.

### 2.5 Local‐scale: Insect identification on experimental plots

Pollinators were identified in situ (see above), whereas frozen Vortis suction samples were identified in the laboratory. To generate a balanced data set with sufficient statistical power within a 1 year identification period, four random Vortis sample replicates were selected for each plant species, and a subset of insect orders were targeted. Targeted orders were chosen on the basis that they included a range of insect functional groups, included species that were mostly >1 mm in length and required relatively modest specialized knowledge to identify. Targeted orders were Blattodea (cockroaches), Coleoptera (beetles), Dermaptera (earwigs), Hemiptera (true bugs), Neuroptera (lacewings) and Orthoptera (grasshoppers and crickets). Individuals were identified to species level wherever possible (88 taxa at species level, genus = 11, subfamily = 2, family = 10, superfamily = 2) and always to a taxonomic resolution that enabled accurate assignment of functional group. Primary works used in insect identification for allocation of functional group (herbivores, omnivores, fungivores/scavengers [detritivores] and predators) can be found in Table S2. Due to our sampling methodology our data do not include any primarily soil inhabiting insects, or any parasitoids.

### 2.6 Geographic‐scale: DBIF summary

The DBIF (Smith & Roy, 2008; Ward, Smith, Pocock, & Roy, 2019) details 60,290 interactions between primarily phytophagous insect species and plants recorded in Great Britain over the last century, based on a wide variety of sources, including entomological journals (e.g. The Entomologists Gazette) and field guides (e.g. Heath & Emmet, 1979). The DBIF interactions represent insect species *x* associated with host plant *y*, rather than standardized abundance information; making it possible to analyse the richness associated with different plant species but not abundance. Nonetheless, the database does include frequency information (numbers of separately recorded interactions between given insect and plant species), meaning that the dissimilarity (distinctiveness) of biotas associated with each plant species could be calculated using Chao–Sorensen ‘abundance’ methods (see below: ‘Geographic‐scale: Statistical analysis’). We refer to DBIF as ‘geographic‐scale’ because the insect–plant data are scattered records from throughout Great Britain (area 209,331 km^2^), but they are ultimately derived from localized observations, although field guide records are commonly derived from many such observations.

### 2.7 Geographic‐scale: DBIF cleaning, native status and range size assignment

We analysed data on ‘higher’ plants (seed plants and ferns), using only insect–plant records that were expertly verified as reliable and included in previous large‐scale analyses (Ward, 1988; Ward, Hackshaw, & Clarke, 1995, 2003; Ward & Spalding, 1993). We only included records that were certain to have occurred in Great Britain, and excluded any records originating from captive breeding studies. In order to enable accurate assignment of host plant native status, arrival date and distribution size we transformed the data set to ensure that all records were at a species level resolution, removing genus level (or above) records (7,362 records; approximately 1/3 of the total), and ‘upgrading’ all subspecies/cultivar/variety information to the species level. BSBI (Botanical Society of Britain and Ireland) taxon version key codes, Stace's *New flora of the British Isles* (2010), UKSI (United Kingdom Species Inventory) codes, the Fauna Europaea (de Jong et al., 2014) and the EPPO Global Database (2019) were used to group together plant and insect species listed under different synonyms.

Plants were classified as neophyte (nonnative, arrived post‐1500), archaeophyte (nonnative, arrived pre‐1500) or native (primarily Holocene colonists). Native status and introduction dates (for neophytes) were assigned to plants from several data sources. Nonnative plant status and neophyte introduction date were sourced from Stace & Crawley's *Alien plants* (2015). PlantAtt (Attributes of British and Irish Plants—Hill, Preston, & Roy, 2004) was used to identify which plants were native, with Stace's *New flora of the British Isles* (2010) confirming 15 additional native plants that were either not included in PlantAtt, or were listed with an uncertain native status. Seventy‐eight plant species could not be classified reliably as native, archaeophyte or neophyte, and so were excluded from the analysis. Also excluded were 19 hybrids. The final data set consisted of 4,397 insect species associated with 679 native plant species, 119 archaeophytes and 234 neophytes.

We quantified host plant range size to account for its well‐established influence on insect species richness (Andow & Imura, 1994; Branco et al., 2015; Brändle & Brandl, 2001; Kennedy & Southwood, 1984; Lawton et al., 1993; Strong et al., 1977). Range size data were provided by O. Pescott, courtesy of the Botanical Society of Britain and Ireland and the Biological Records Centre. Range size was quantified as the number of hectads (10 × 10 km grid squares) that a plant was recorded in between 1987 and 1999 (within Great Britain including the Isle of Man—vice counties 1–112), which represented a period of intensive recording for the New Atlas project (see Pescott, Humphrey, & Walker, 2018 for further information on BRC plant records). We did not include Irish or European plant records as the majority of insect dispersal occurs within Great Britain (for example, the range size of most British butterfly populations is limited to within Britain—Asher et al., 2001). Plants with no recorded range size information (i.e. species too rare to be detected in the specified period) were assigned a range size value of zero (16 plant species).

### 2.8 Local‐ and geographic‐scale: Host plant phylogenetic relationships

Phylogenetic relationships between plants were trimmed from a recently published global phylogeny of vascular plants (Qian & Jin, 2016), using the R package pez (Pearse et al., 2015), producing three custom phylogenies (appropriate for the analyses of local‐scale pollinators, local‐scale Vortis and geographic‐scale DBIF data respectively). In cases where species were not found in the phylogeny all members of their clade were replaced with a polytomy (local‐scale pollinators = 31% of species not found, local‐scale Vortis = 33%, geographic‐scale DBIF = 17%). 11 plant species could not be assigned a place in the DBIF phylogeny as they belonged to clades not included in Qian and Jin's megaphylogeny, and thus were excluded from any analysis involving host plant phylogenetic isolation. Four phylogenetic isolation measures were calculated:

1. *Mean phylogenetic isolation*: The mean divergence time (in millions of years) from a plant to every other plant in the phylogeny.
2. *Nearest phylogenetic neighbour distance*: The divergence time from a plant to its closest relative in the phylogeny.
3. *Mean phylogenetic isolation from natives*: The mean divergence time from a *nonnative* plant to every other *native* plant in the phylogeny.
4. *Nearest native phylogenetic neighbour distance*: The divergence time from a *nonnative* plant to its closest *native* neighbour in the phylogeny.

### 2.9 Local‐scale: Statistical analysis

Insect taxa were identified to varying taxonomic resolutions, as detailed above. Consequently, richness values represent taxon richness, as opposed to species richness. There were several cases where two taxa in the Vortis data were present on the same plant species but could not be fully distinguished (e.g. Anthocoridae nymph vs. Anthocoris nemorum). In these instances all recorded individuals contributed to total values of abundance on a plant. However, during calculation of richness potentially overlapping taxa contributed only once to the total taxon richness associated with a plant.

The Vortis and pollinator data were in all instances analysed separately, given the different methodologies and that the two sampling protocols were carried out several years apart (2010–2013 vs. July 2016), with a few plant species replacements taking place during the interim period (see Supporting Information). Insect richness and abundance values represent the summed richness and abundance found on all replicates of each plant species (grouping plots and sites) to reduce zeros and low sample sizes. This was appropriate as plant species plot locations were randomized within sites and balanced across the two sites (which were ~154 m apart and shared the same soil type).

Although the design was balanced (this balance was maintained for the Vortis analysis following randomized subsampling, as described above), different numbers of plant individuals, quantities of flower per plant and duration of flowering meant that we needed to control for this source of variation in the pollinator analysis. Flowering units (amount of flower) represented the mean of all replicates of a species and was included in the analysis. A Julian date was also calculated for each sampling event (number of days from 1 January). The median of all sampling Julian dates was included as a measure of phenology for each plant species. Finally, the log of the number of replicates of each plant species was included as a predictor in all pollinator analyses, to account for sampling effort effects stemming from large variation in replication (mean = 72.1 samples per plant species, median = 62, but this ranged from 2 to 307 replicates). Despite even sampling within the Vortis data plant architectures varied, and so we included both the median volume (area*height) of each plant species and host plant branching architecture in the analysis.

Nine Vortis insect taxa were excluded prior to the calculation of community distinctiveness. These taxa were identified to a coarse taxonomic resolution, which precluded their distinction from other taxa resolved to a finer level (e.g. Anthocoridae nymph vs. Anthocoris nemorum). All pollinator taxa were included for calculation of community distinctiveness, as despite varying taxonomic resolution all taxa could be distinguished. Community distinctiveness was quantified in the following way for the Vortis and pollinator data. A pairwise dissimilarity matrix of the insects associated with all plants (that hosted an insect species richness >0) was created using the Chao–Sorensen abundance‐based dissimilarity index, as our data contained a substantial fraction of scarcely abundant species, and classic Jaccard and Sørensen indices often perform poorly in these situations (Chao, Chazdon, Colwell, & Shen, 2005). The Chao–Sorensen dissimilarity matrix was reduced using nonmetric multidimensional scaling (NMDS), which collapses information from multiple dimensions into a few, allowing the data to be more easily visualized and interpreted (Kruskal, 1964). NMDS collapsed the Chao–Sorensen matrix into three dimensions with stress values of less than 0.2 for both the Vortis (stress = 0.161) and pollinator (stress = 0.165) data, indicating a good representation of the data in the reduced dimensions. Finally, the distance was measured from each plant's location in three‐dimensional space to the group centroid (co‐ordinates 0, 0, 0). This distance represented each plant's value of insect community distinctiveness. This technique has been adapted from similar approaches used to calculate mean β‐diversity across a group of sites (by taking the mean distance from each site to the group centroid in NMDS space; Anderson, Ellingsen, & McArdle, 2006; Myers, Chase, Crandall, & Jiménez, 2015). The location of each host plant in three‐dimensional NMDS space is presented in Figures S3 and S4.

The mean levels of insect host specialization were contrasted between the two data sets (Vortis and pollinator) via the *d*′ index (Blüthgen, Menzel, & Blüthgen, 2006). The number of interactions that an entity (insect or plant) had with all other available partners (expressed as the proportion of observed links out of those possible) is used when calculating *d*′. Thus, *d*′ can be interpreted as the deviation of an insect's actual interaction frequencies from a null model which assumes that all plant partners were used in proportion to their availability. Possible *d*′ values range from 0 (perfect generalist) to 1 (perfect specialist).

Vortis insect and pollinator nearest phylogenetic neighbour distance models did not include host plant native status as a predictor because, by definition, natives and congeners within a plant species triplet were almost always congeneric, whilst exotics were always more distantly related. This meant that native status was in effect a categorical approximation of phylogenetic proximity.

We considered statistical associations between predictor variables, but these were generally weak (Kendall Tau‐b correlation tau <0.4) or absent in the pollinator and Vortis models (Supporting Information). Status had a significant effect on host plant median Julian date in the pollinator models (χ^2^ = 9.21, *p* = .010, *df* = 2), however, median Julian date did not significantly improve the overall pollinator models, and so was not included in the final analysis.

### 2.10 Geographic‐scale: Statistical analysis

Values of insect richness represented the summed richness from all sources reporting on a plant species. The log of the number of sources reporting on each plant species was included as a predictor in all analyses, to account for sampling effort effects stemming from large variation in the number of sources (1–64 sources, mean = 6, median = 3, where a source was defined as an individual article).

Insect community distinctiveness was defined as the Chao–Sorensen abundance‐based dissimilarity (we employed the Chao–Sorensen index as the DBIF data contained a high proportion of scarcely abundant species; Chao et al., 2005) between the insect community on a given nonnative host and the entire insect pool collectively found on well‐sampled native plants (insect richness ≥10) within the DBIF all grouped together (the very large variation in insect richness among host plants meant that the NMDS method used for the local‐scale analyses could not converge in three‐dimensional space for the DBIF data). Only plants that hosted an insect richness ≥10 were included (natives = 206 plant species, archaeophytes = 30, neophytes = 26), ensuring that host plants had been sufficiently sampled for dissimilarity analysis. Qualitatively similar results were obtained when the analyses were repeated with the insect pool found on all native plant species.

We accounted for variation in sampling effort (log of the number of literature/data sources reporting insect species on a plant) in all DBIF analyses because this was a strong predictor of insect richness (Table S6). We also considered associations among predictor variables in the DBIF data (see Supporting Information). Sampling effort was weakly correlated (Kendall Tau‐b correlation tau <0.4) with nonnative host plant mean phylogenetic isolation from natives, host plant range size and neophyte introduction date. Host plant range size and neophyte introduction date were also weakly correlated. Host plant native status was significantly associated with all DBIF model predictors. A potential implication of these associations is considered in Section 3.

### 2.11 Local‐ and geographic‐scale: Statistical modelling frameworks common to both scales

All statistical analyses were carried out in R (R Core Team, 2017) using R Studio (RStudio Team, 2016). See Supporting Information for full a list of R packages used. The distributions and nature of data varied somewhat between analyses, resulting in slightly different model formulations.

We used either Poisson or negative binomial regression (depending on data overdispersion, both specified with a log link) for the effects of plant native status, phylogenetic isolation, neophyte arrival date (DBIF only) and range size (DBIF only) on insect community richness and abundance (local‐scale only) and beta regression (specified with a log link) to test the effect of all of the above predictors on insect community distinctiveness. Status contrasts were calculated with post hoc Tukey tests. Only beta regression mean test values are reported in this manuscript: the beta regression mean submodel reports the influence of regressors on the mean of a dependent variable, whereas the beta regression precision submodel quantifies the effect of model regressors on dependent variable dispersion (Cribari‐Neto & Zeileis, 2010). Models were constructed via addition of predictors of interest, and comparison of models with likelihood ratio tests and AIC values. Good model fit was determined via inspection of diagnostic plots, and via calculation of *D* ^2^/pseudo *R* ^2^ values. *D* ^2^ is the glm equivalent of *R* ^2^, and represents the proportion of deviance explained by a Poisson or negative binomial model (Guisan & Zimmermann, 2000), whilst pseudo *R* ^2^ is the beta regression equivalent of *R* ^2^ (McFadden, 1973). Predictors included in the best fitting models are detailed in Section 3.

Estimating deviance contributions was complicated by the consistently large effect of sampling effort in two of our analyses (local‐scale pollinators = log(replicates), DBIF = log(sources)), so we calculated two measures that incorporated the type I and type II SS (Herr, 1986) explained by our predictors of interest.

1. A minimum estimate of the deviance (*D*) explained by all other predictors after accounting for sampling effort:
  (1) $$D=\frac{\text{type II deviance of all other predictors}}{\text{null deviance}-\text{type II deviance of sampling effort}}$$
2. A maximum estimate of the deviance (*D*) explained by all other predictors after accounting for sampling effort:
  (2) $$D=\frac{\begin{array}{c} \text{type I deviance of all other predictors}\\ +\;\text{type II deviance of all other predictors} \end{array}}{\text{null deviance}-\text{type II deviance of sampling effort}}$$

The type II deviance explained by a predictor was calculated using the ANOVA function in R (Fox & Weisberg, 2019). Type II deviance represents the deviance uniquely explained by a predictor, and type I equates to the deviance shared by a predictor with others. Thus, in Equation (2) the dividend represents the maximum amount of deviance that may have been explained by our predictors of interest, and the divisor is the deviance that remains in the model after accounting for the deviance uniquely explained by sampling effort. We calculated the dividend in Equation (2) as follows:

(3) dividend=null deviance−residual deviance−type II deviance of sampling effort

A minimum and maximum estimate of explained deviance were calculated for all Poisson and negative binomial models (insect species/taxon richness and abundance), but were not calculated for beta models (insect community distinctiveness) as beta regression does not have all of the properties of ‘classical’ GLMs (Cribari‐Neto & Zeileis, 2010), and so reliable calculation of type II SS was not possible.

### 2.12 Local‐ and geographic‐scale: Rarefaction

We created sample‐based richness rarefaction curves (methods adapted from Colwell et al., 2012) to evaluate how the diversity of insects associated with native species (pooled) differed from the accumulation of diversity on other categories of plant (e.g. neophytes pooled). We estimated rarefaction confidence intervals by bootstrapping 10,000 times, with a sample classified as a plant replicate for the local‐scale pollinator or Vortis data, and as a unique data source (normally a single article or other publication) and plant species combination for the geographic‐scale DBIF data.

We also implemented a ‘combined’ rarefaction to represent the accumulation of richness in a mixed community. For the local‐scale data, this mixed line displays the rarefaction of the total richness found on all plant replicates, but the geographic‐scale DBIF mixed line equalizes the number of sources from plants of different native status. Thus, the DBIF line represents a summary of the rarefaction of 200 random samples composed of 1/3 natives, 1/3 archaeophytes and 1/3 neophytes, with each individual rarefaction bootstrapped 10,000 times, and the upper and lower confidence intervals of the mixed line representing the maximum and minimum 95% confidence intervals from the rarefaction of the 200 random samples.

## 3 RESULTS

The three data sets and the hypotheses tested with them are summarized in Table 1. Pollinators were sight‐recorded from the experimental plots for 23 native plant species (1,939 replicates; pollinator replicates were comprised of plant individuals recorded in different years/seasons), 21 congeners (1,390 replicates) and 20 exotics (1,358 replicates); giving 6,307 individual insects from 54 taxa. Plant individuals/patches (replicates) were Vortis (suction) sampled from the experimental plots for 14 native plant species (total 56 replicates), 13 congeners (52 replicates) and 15 exotics (60 replicates); capturing 2,071 individual insects representing 108 taxa of mixed trophic and functional groups. Within the DBIF geographic‐scale database 4,397 insect and mite species were reported interacting with 679 native, 119 nonnative archaeophyte and 234 nonnative neophyte plant species.

**Table 1:** An overview of the three data sets

	Local‐scale pollinators	Local‐scale Vortis	Geographic‐scale DBIF
Sampling methodology	Pollinators sampled 2010–2013 (March–September) Eight minutes per plot Pollinators identified on the wing to as close to species level as possible	Vortis suction sampling of plant inhabiting insects (July 2016) Thirty seconds per plant Insects identified with keys to as close to species level as possible	Database detailing interactions reported in both primary and secondary literature (from 1920 onwards)
Native status	Native Congeneric nonnative Exotic nonnative	Native Congeneric nonnative Exotic nonnative	Native Archaeophyte (arrival pre 1,500) Neophyte (arrival post 1,500)
No. of plant species	Total = 64 Native = 23 Congeneric nonnative = 21 Exotic nonnative = 20	Total = 42 Native = 14 Congeneric nonnative = 13 Exotic nonnative = 15	Total = 1,033 Native = 679 Archaeophyte = 120 Neophyte = 234
No. of insect taxa	54	108	4,397
No. of insect individuals	6,307	2,071	NA
Predictors of interest	Phylogenetic isolation Native status	Phylogenetic isolation Native status	Phylogenetic isolation Native status Range size Neophyte arrival date
Controls	Median flowering units Median Julian date Log no. of replicates	Median volume Branching architecture	Log no. of sources
Hypotheses tested	a–d (see Section 1) The experimental plots contained garden plants, and so it was not possible to include time since host plant introduction and host plant range size in the analysis	a–d (see Section 1) The experimental plots contained garden plants, and so it was not possible to include time since host plant introduction and host plant range size in the analysis	a + c–f (see Section 1) The DBIF data contained primarily herbivores (99% of records), and so it was not possible to include functional/trophic group in the analysis

We found no evidence that insect functional/trophic groups (herbivores, detritivores, omnivores and predators) responded differently to host plant native status and phylogenetic isolation within the Vortis samples (Table S3), so all Vortis insects were grouped together for the analyses presented in the following text.

### 3.1 Insect abundance: Local‐scale

**Figure 1:**
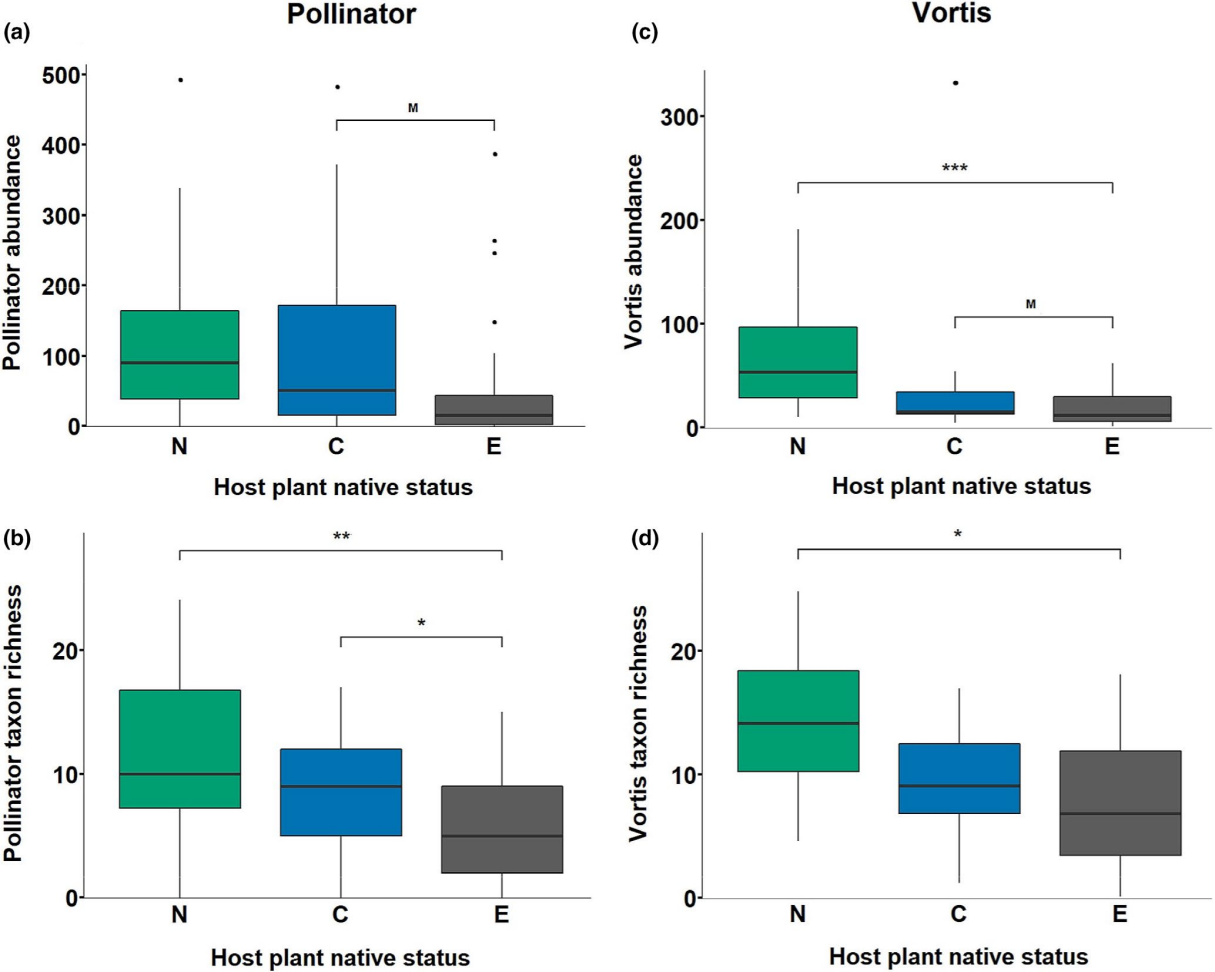
Local‐scale insect taxonomic richness and abundances associated with N = native, C = congener, and E = exotic plant species. Boxplots represent median, interquartile range, and 1.5× the interquartile range. Points represent outliers. Significance of Tukey post hoc contrasts M = ‘marginal’ *p* of .05 < .1, *≤.05, **≤.01, ***≤.001. *D* ^2^ represents the proportion of deviance explained by a model. *D* represents the range of deviance explained by all predictors of interest, after accounting for sampling effort (log(Replicates)) in pollinator models. See Section 2 for an explanation of the calculation of *D*, and of the model building process. (a) Pollinator abundance. Negative binomial model (Pollinator Abundance  ~ log(Replicates) + Flowering Units + Status) *D* ^2^ = 0.624, *D* = 0.280–0.286. Sample size of *N* = 22 plant species, C = 21, E = 20. (b) Pollinator taxon richness. Negative binomial model (Pollinator Taxon Richness ~ log(Replicates) + Flowering Units + Status) *D* ^2^ = 0.636, *D* = 0.160–0.264. Sample size of *N* = 22 plant species, C = 21, E = 20. (c) Vortis insect abundance. Negative binomial model (Vortis Insect Abundance ~ Status) *D* ^2^ = 0.205. Sample size of *N* = 14 plant species, C = 13, E = 15. (d) Vortis insect taxon richness. Negative binomial model (Vortis Insect Taxon Richness ~ Status) *D* ^2^ = 0.163. Sample size of *N* = 14 plant species, C = 13, E = 15

The highest insect abundances (total number of insect individuals per plant species, measured by Vortis suction samples and pollinator observations) were associated with native plant species (and the lowest abundances with exotic plants; Figure 1a,c; Table S4). Vortis abundance was significantly lower on exotic plants compared to native plants, with the median abundance on exotics being 28% of that on native plant species (Figure 1c; Table S4). Median pollinator abundance on exotics was 18% of that on native plant species, but outliers meant that there was no significant difference (Figure 1a; Table S4). Median abundances for nonnative congeners were intermediate between that of native species and exotics for both Vortis samples and pollinators (Figure 1). Nonnative congeners supported marginally higher (.05 < *p* < .1 in post hoc Tukey tests) pollinator and Vortis sample abundances than the corresponding exotic plants (Figure 1a; Table S4).

We included additional predictor variables in our models of insect abundance to account for host plant structural characteristics (flowering units per plant for pollinators, and plant volume and branching architecture for Vortis insects), sampling effort (pollinators only—log no. of replicates) and sampling date (pollinators only—due to differences among plant species in their flowering phenologies). Pollinator abundance increased with sampling effort and the number of flowering units per plant species (Table S4). In contrast, sampling date (median Julian sampling day of each plant species) did not lead to significant improvement of the best model (likelihood ratio test χ^2^ = 1.30, *p* = .255, *df* = 1), and so was excluded from this and subsequent pollinator abundance models. Sampling dates and replication were fully balanced for Vortis samples (so not included in models), but plant species did differ in their size and architecture. However, neither estimated median plant volume (χ^2^ = 0.61, *p* = .435, *df* = 1) nor branching architecture (χ^2^ = 0.74, *p* = .389, *df* = 1) significantly improved the model, and hence plant volume and branching architecture were excluded from all Vortis abundance models.

**Figure 2:**
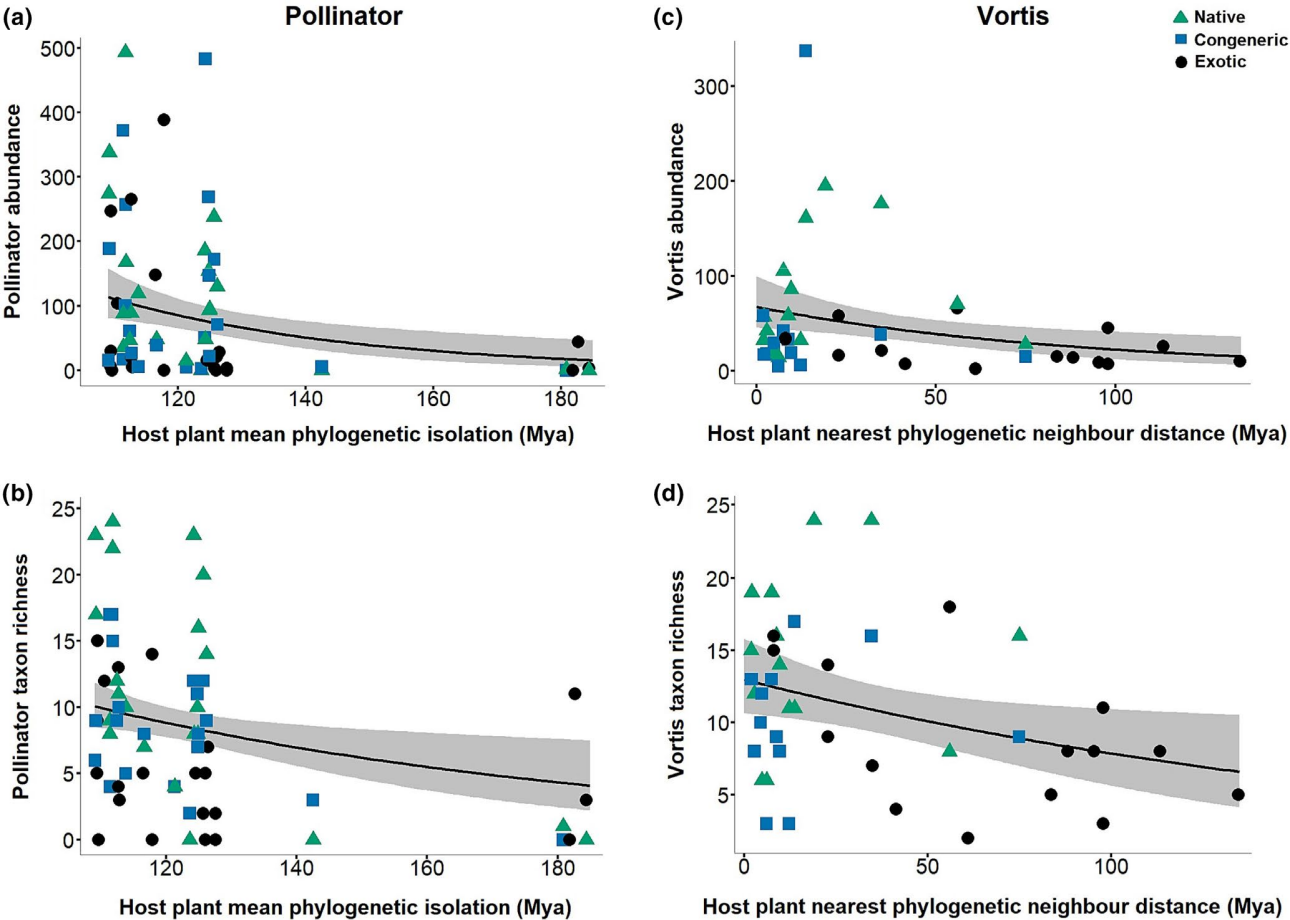
The effect of host plant phylogenetic isolation on local‐scale pollinator and Vortis insect abundance and taxon richness. Partial regression plots display the effect of our focal predictor (phylogenetic isolation), whilst holding all other predictors at their mean. Shaded areas represent 95% confidence intervals. Data points represent individual plant species. Mean phylogenetic isolation (MPI) = mean distance in millions of years from host plant to all other plants in the local community. Nearest phylogenetic neighbour distance (NPN) = distance in millions of years from host plant to closest phylogenetic neighbour in the local community. See Section 2 for details of the calculation of *D* ^2^ and *D*. (a) Pollinator abundance. Negative binomial model (Pollinator Abundance ~ log(Replicates) + Flowering Units + MPI) *n* = 64, *p*(MPI) = 0.002, *D* ^2^ = 0.632, *D* = 0.209–0.381. (b) Pollinator taxon richness. Negative binomial model (Pollinator Taxon Richness ~ log(Replicates) + Flowering Units + Status + MPI) *n* = 64, *p*(MPI) = 0.010, *D* ^2^ = 0.671, *D* = 0.154–0.449. (c) Vortis insect abundance. Negative binomial model (Vortis Insect Abundance ~ NPN) *n* = 42, *p*(NPN) = 0.005, *D* ^2^ = 0.108. (d) Vortis insect taxon richness. Negative binomial model (Vortis Insect Taxon Richness ~ NPN) *n* = 42, *p*(NPN) = 0.017, *D* ^2^ = 0.112

Native status (native, nonnative congener, or exotic) is still a relatively coarse categorical variable, whereas the phylogenetic isolation (measured in millions of years since divergence between plant species) of a nonnative congener or exotic may better function as a proxy for the novelty of a nonnative plant habitat, from the perspective of potential insect colonists. Abundances of associated insects declined with phylogenetic isolation for both the pollinator and Vortis samples (Figure 2a,c; Table S4), indicating that more divergent plant habitats, on average, support lower insect abundances. However, exact details differed for the two datasets. Pollinators were influenced by host plant relationships with the entire experimental community (mean phylogenetic isolation; Figure 2a), whereas insects sampled by Vortis were influenced by host plant relationships with their closest phylogenetic neighbour (nearest phylogenetic neighbour distance; Figure 2c). A similar pattern emerged when the phylogenetic isolation of nonnative plants from *native* species in the plots was considered, although these effects were only marginally significant (Table S4). Overall, these results suggest a broader range of host plant sources for pollinators than for other plant‐associated insects.

### 3.2 Insect taxonomic richness: Local‐scale

Insect taxon richness (based on pollinator observations and Vortis samples) was highest on native plants and lowest on exotic plants, while the richness associated with nonnative congeners was intermediate, particularly in the pollinator samples (Figure 1b,d; Table S5). For pollinators, the log of the number of host plant replicates and the number of flowering units were retained as strong predictors in the best models. However, sampling date did not lead to significant improvement of the pollinator richness model (χ^2^ = 0.13, *p* = .718, *df* = 1), and so was excluded from this and subsequent models. For Vortis samples, neither host plant median volume (χ^2^ = 0.13, *p* = .717, *df* = 1) nor branching architecture (χ^2^ = 0.11, *p* = .745, *df* = 1) significantly improved the best model, and thus were not included in any statistical models of Vortis insect richness.

**Figure 3:**
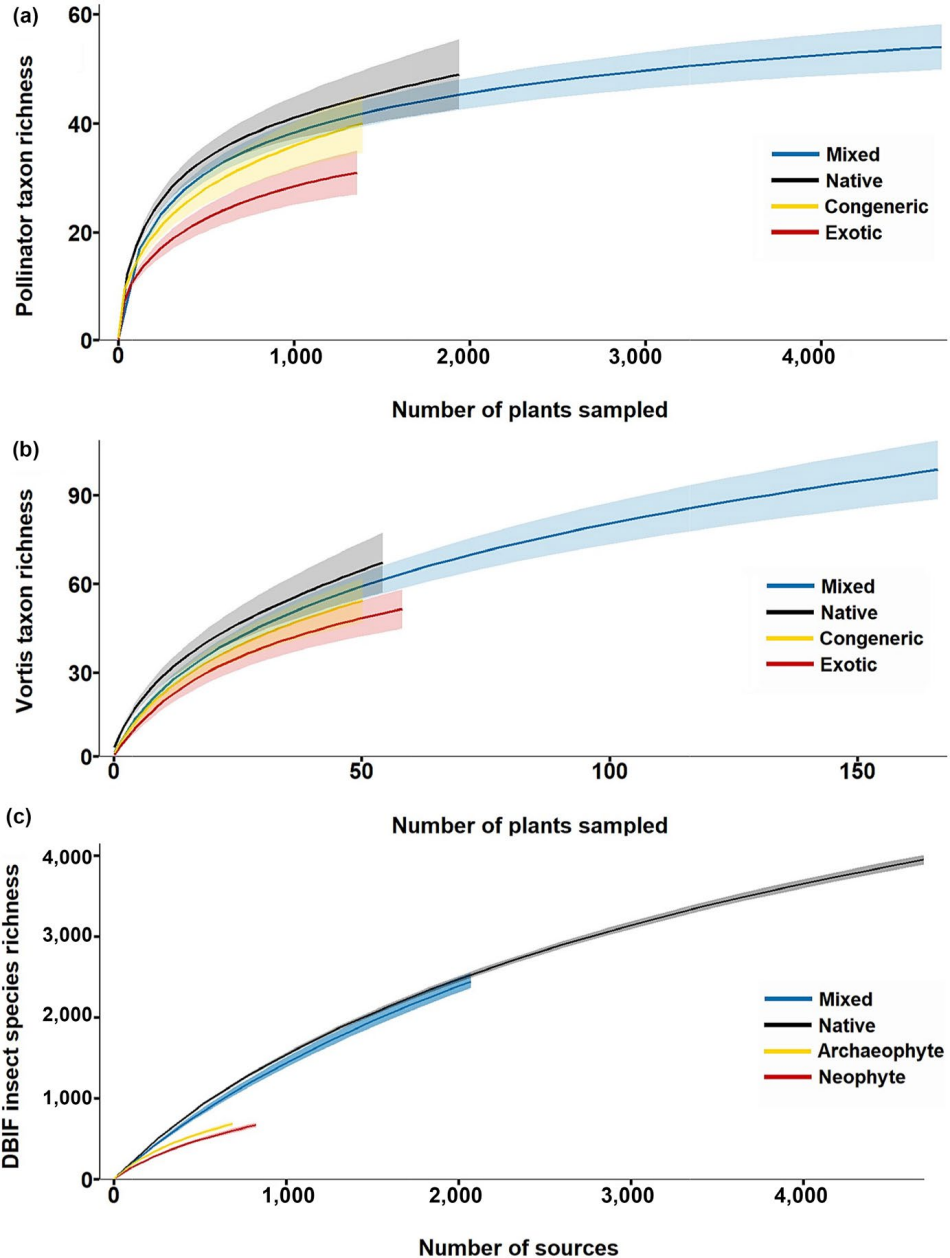
Sample‐based rarefaction of the local‐scale pollinator (a), local‐scale Vortis (b) and geographic‐scale DBIF data (c). Shaded areas represent 95% confidence intervals, bootstrapped 10,000 times for each plant type. Vortis and pollinator mixed lines represent rarefaction of the entire data set. The exception is in (c), where the DBIF mixed line represents the summary of the rarefaction of 200 random samples composed of 1/3 natives, 1/3 archaeophytes and 1/3 neophytes; the upper bound is the minimum of the 200 upper 95% confidence intervals, and the lower bound is the maximum of the 200 lower 95% confidence intervals. A local‐scale sample was defined as a plant species replicate. A DBIF sample was defined as a unique source and plant species combination. Pollinator: sample size of native = 1,941 samples (plant*date*year), congener = 1,390, exotic = 1,358, mixed = 4,689. Vortis: sample size of native = 56 samples (plant), congener = 52, exotic = 60, mixed = 168. DBIF: sample size of native = 4,700 samples (source*plant), archaeophyte = 691, neophyte = 826, mixed = 2,073

Sample‐based (number of plant individuals) rarefaction analyses confirmed the significant differences in taxon richness, as shown by the nonoverlapping confidence intervals of the curves for exotic and native plants in Figure 3a,b. Richness was significantly reduced on exotic plants, compared to natives, whilst nonnative congeners were again intermediate.

The richness of associated insect taxa also declined with the phylogenetic isolation of host plants for both the pollinator and Vortis samples (Figure 2b,d; Table S5). As for abundance, the richness of pollinators on a plant species was influenced by its isolation from the entire plant assemblage (mean phylogenetic isolation; Figure 2b), and Vortis insect richness was only influenced by host plant isolation from the most closely related other plant species (nearest phylogenetic neighbour distance; Figure 2d). Unlike for abundance, pollinator richness was also impacted by the most closely related other plant (nearest phylogenetic neighbour distance; Figure S5a). Similar effects emerged when the phylogenetic isolation of nonnative plants from native species was considered (Figure S5b; Table S5), although these effects were mostly nonsignificant.

### 3.3 Insect taxonomic richness: Geographic‐scale

**Figure 4:**
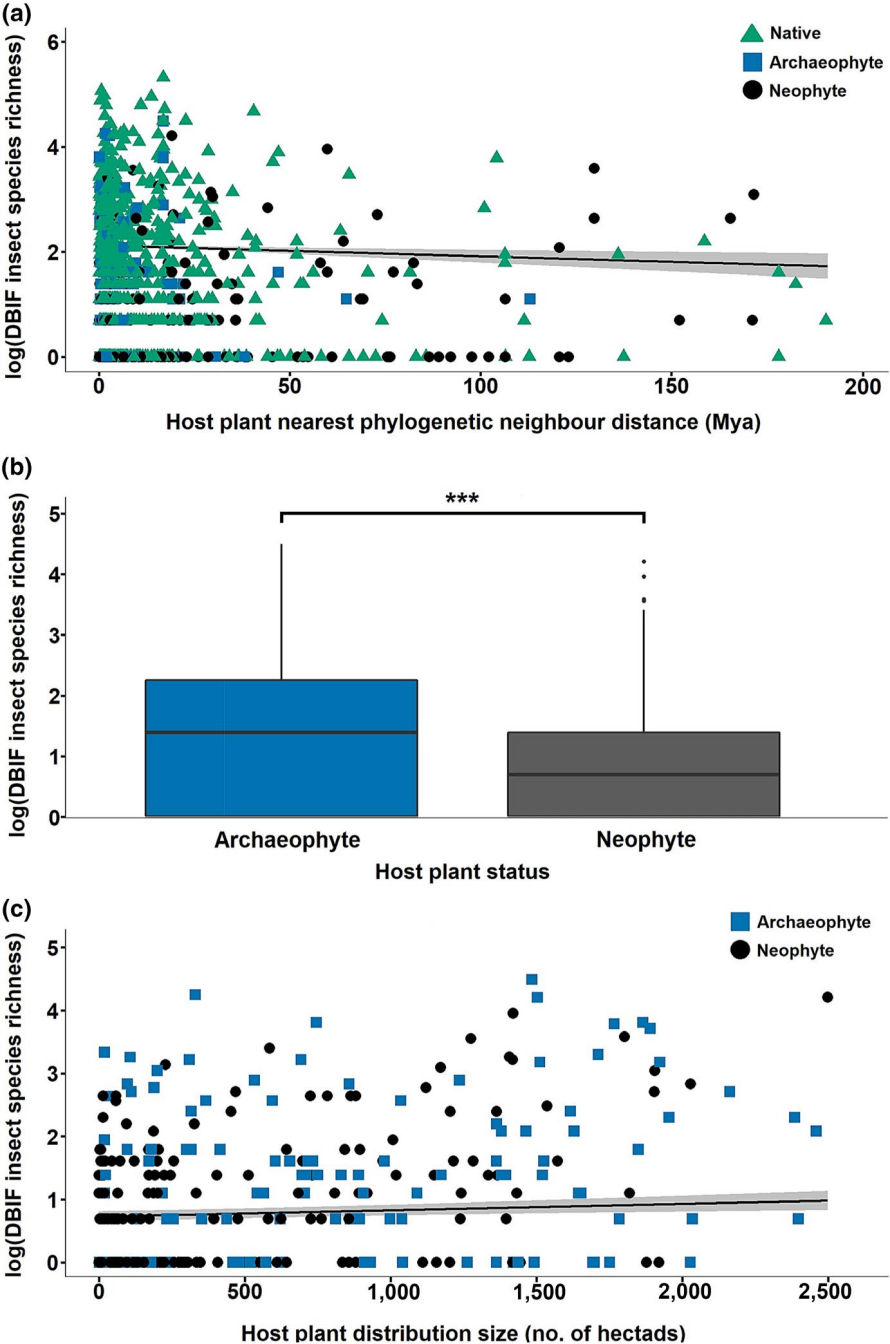
The effect of host plant phylogenetic isolation, native status, and range size on geographic‐scale DBIF insect species richness. Partial regression plots (a) and (c) display the effect of our focal predictors (phylogenetic isolation and range size), whilst holding all other predictors at their mean. Shaded areas represent 95% confidence intervals. Data points represent individual plants species. Boxplots (b) represent median, interquartile range and 1.5× the interquartile range. Boxplot points represent outliers. Asterisks denote significance of Tukey post hoc contrasts (***p < .001). Nearest phylogenetic neighbour distance (NPN) = distance in millions of years from host plant to closest phylogenetic neighbour in the DBIF. See Section 2 for details of the calculation of *D* ^2^ and *D*. (a) Negative binomial model (Richness ~ log(Sources) + NPN + Hectads) *n* = 1,022, *p*(NPN) = 0.002, *D* ^2^ = 0.924, *D* = 0.010–0.829. (b) Poisson model (Richness ~ log(Sources) + Status + Hectads) *n* = 352, *p*(Status) <1e‐04, *D* ^2^ = 0.927, *D* = 0.025–0.763. (c) Negative binomial model (Richness ~ log(Sources) + Status + Hectads) *n* = 352, *p*(Hectads) = 0.002, *D* ^2^ = 0.927, *D* = 0.025–0.763

Rarefaction analyses showed that richness accumulated (with increased sampling effort) at a significantly reduced rate on introduced plants (neophytes and archaeophytes) compared to natives, and that archaeophytes accumulated species at a faster rate than neophytes (Figure 3c). Statistical modelling revealed that the species richness of insects increased significantly when introduced plants were closely related to native plant species (for three of the four metrics of phylogenetic isolation), increased significantly with the range sizes of the introduced plants and increased with DBIF sampling effort (Figure 4; Figure S6; Table S6). Date of introduction (for neophyte‐only models) had no significant effect on insect species richness (Table S6). Host plant native status (neophytes introduced since 1500, archaeophytes introduced prior to 1500) had a highly significant effect on the richness of the insects found on nonnative plants in the DBIF (Figure 4; Table S6), with archaeophytes hosting more insect species than neophytes. The inclusion of native status in our models led to the loss of the significant effects of two of our four metrics of phylogenetic isolation (nonnative plant mean isolation from natives, and nonnative plant nearest native neighbour distance). The association of DBIF host plant native status with all other model predictors (see Supporting Information) was the probable cause of this loss of significance.

It was difficult to determine the ‘true’ deviance (effect sizes) explained by phylogenetic isolation, range size and native status because of the large effect of sampling effort (which varies greatly among plant species in the DBIF), and because there were large overlaps in the deviance which could be explained by sampling effort and the other predictors. The strong influence of sampling effort (sources) is evident when comparing *z* values: phylogenetic isolation *z* = −2.99 to −3.06; range size *z* = 2.70–5.54, native status (neophyte/archaeophyte) *z* = −3.06 to −4.14; log(sources) *z* = 44.70–69.62. After accounting for sampling effort (see Section 2), the deviance explained by geographic range size, phylogenetic isolation and/or host plant native status ranged from a minimum of *D* = 0.007–0.025 (assuming that all shared deviance was explained by sampling effort) to a maximum of *D* = 0.758–0.830 (assuming that all shared deviance was explained by the predictor variables of interest).

### 3.4 Specialization and community distinctiveness: Local‐scale

**Figure 5:**
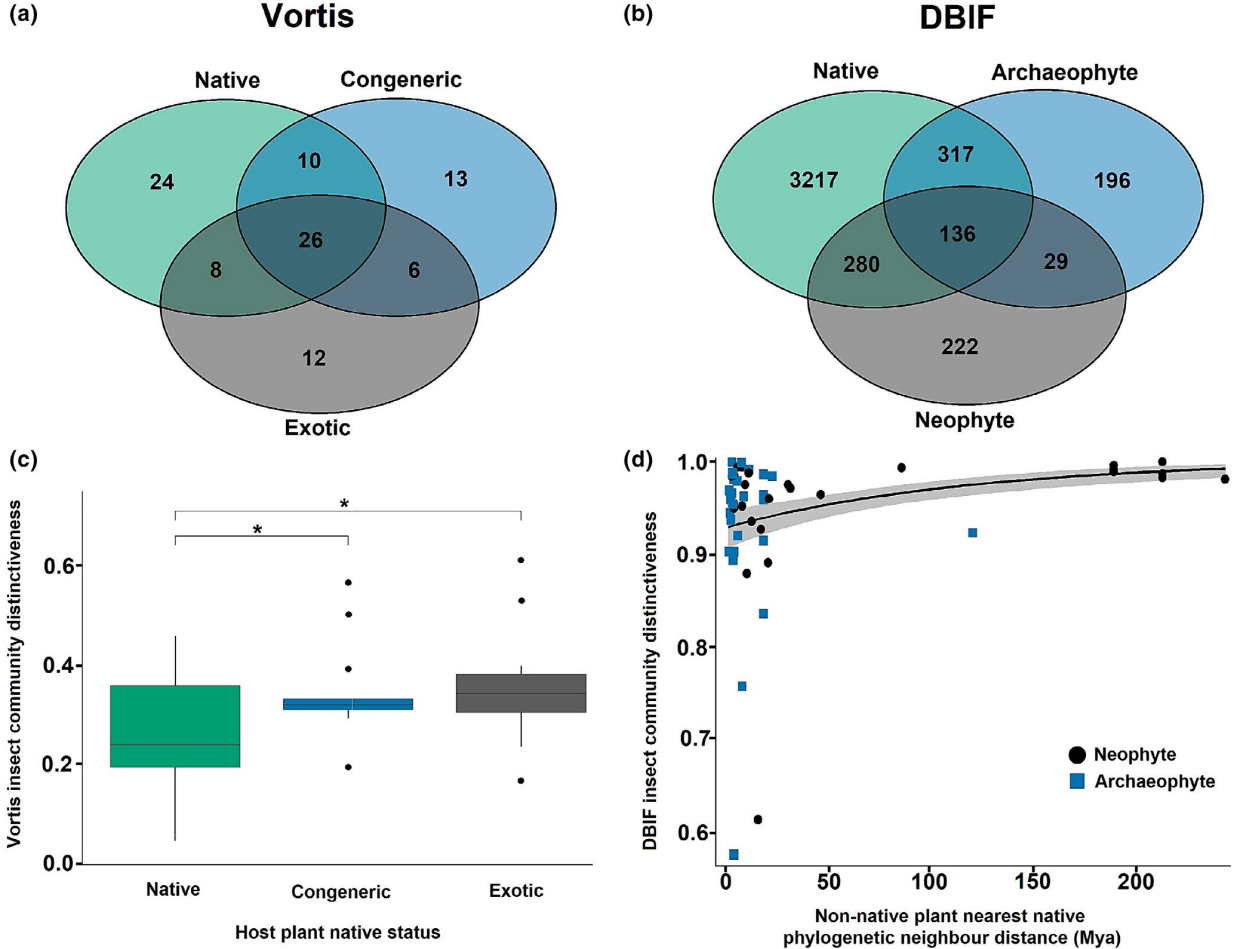
The effect of host plant native status and phylogenetic isolation on insect community distinctiveness. Distinctiveness was bounded between 0 and 1. See Section 2 for details of the calculation of pseudo *R* ^2^, and for the distinction between beta regression mean and precision submodels. (a) Venn diagrams displaying the number of local‐scale Vortis taxa unique to, and shared between each host plant native status. Sample size of native = 14 plant species, congeneric = 13, exotic = 15. (b) Venn diagrams displaying the number of geographic‐scale DBIF species unique to, and shared between each host plant native status. Sample size of native = 679 plant species, archaeophyte = 120, neophyte = 234. (c) Local‐scale Vortis insect community distinctiveness on the different host plant statuses. Vortis insect distinctiveness was calculated using a NMDS approach (see Section 2). Boxplots represent median, interquartile range and 1.5× the interquartile range. Boxplot points represent outliers. Asterisks denote significance of Tukey post hoc contrasts (*p < .05). Beta model (Vortis Insect Community Distinctiveness ~ Status) pseudo *R* ^2^ = .182. Sample size of Native = 14 plant species, Congeneric = 13, Exotic = 15. (d) Geographic‐scale DBIF insect community distinctiveness with increasing host plant phylogenetic isolation. A partial regression plot displays the effect of our focal predictor (phylogenetic isolation), whilst holding all other predictors at their mean. Shaded areas represent 95% confidence intervals. Data points represent individual plant species. Nonnative host plant nearest native phylogenetic neighbour distance (NPNN) = distance in millions of years from *nonnative* host plant to closest *native* phylogenetic neighbour in the DBIF. The distinctiveness of the insect community on a plant was represented by dissimilarity from the pool of insects found on native plants (see Section 2). Beta model (DBIF Insect Community Distinctiveness ~ log(Sources) + NPNN|NPNN) *n* = 56, *p*(NPNN) <1e‐04, pseudo *R* ^2^ = .218

Pollinator taxa were relative generalists, and were associated with a higher proportion of available plant species compared to the more specialized Vortis‐sampled insect groups (*d*′ specialization index values: mean Vortis *d*′ = 0.41 vs. mean pollinator *d*′ = 0.26; Wilcoxon signed rank test: *W* = 1,131, number of pollinators = 54, number of Vortis = 100, *p* < 1e‐04). Consequently, a higher proportion of pollinator taxa (46%) were shared between plants of the three native status than was the case for the Vortis samples (26%; Figure 5a; Figure S7). Recall that the abundance and richness of Vortis insects were predicted by phylogenetic isolation from the most closely related plant, whereas pollinators were also influenced by phylogenetic isolation from all other plants in the experimental plots (Figure 2; Tables S4 and S5; Figure S5). Thus, specialized insect faunas may be acquired primarily from similar, closely related sources, whereas the abundances and richness of generalists may depend on the wider plant community.

Exotic plants and congeners supported significantly more distinctive pollinator and Vortis communities than natives (Figure 5c; Figure S8; Table S7). Interestingly, 32% of the insect taxa in the Vortis data were only sampled from the nonnative plants: either congeners or southern hemisphere exotics (Figure 5a; compared to 9% of the more generalized pollinator taxa—Figure S7). The presence of species uniquely sampled from nonnative plants likely explains why a mixed community (composed of all the plants on the plots) accumulated richness at a rate comparable to that of a community of native plants alone (rarefaction analysis—Figure 3a,b), despite the higher average richness of individual native plant species.

### 3.5 Community distinctiveness: Geographic‐scale

Nonnative plants that were phylogenetically isolated from native plants supported the most distinctive insect communities (Figure 5d; Figure S9; Table S8). This was a highly significant and moderately sized positive effect (phylogenetic isolation *z* = 5.05–7.51, log(sources) *z* = −2.67 to −3.02). There were no significant effects of range size (number of hectads in Great Britain), neophyte versus archaeophyte status, or neophyte arrival date on insect community distinctiveness (Table S8).

Around 10% of DBIF insect taxa were only sampled from nonnative plants: either archaeophytes or neophytes (Figure 5c). The presence of species unique to nonnative plants can be clearly seen in the sample‐based rarefaction of the DBIF data, where a modelled mixed landscape of 1/3 natives, 1/3 archaeophytes and 1/3 neophytes would be expected to host a comparable number of species at the reference sample size (2,073) to a landscape composed purely of natives (Figure 3c), despite archaeophytes and neophytes accumulating insect species at a slower rate than natives.

## 4 DISCUSSION

Together, the results indicate that novel plant habitats that share some similarities with long‐standing ‘native’ plant habitats accumulate higher abundances and diversities of associated insect species compared with novel plant habitats that are more distinct. In this regard, plant origin (native, congeneric nonnative and exotic) and phylogenetic isolation are alternative proxies for the distinctive phenotypes of introduced plants that determine their suitability as habitats for insects, following their arrival in a new location. Thus, the results all point towards introduced plants accumulating more abundant and more diverse (species/taxon rich) communities of insects when they share some attributes (congeneric, low phylogenetic isolation; and hence an increased likelihood of chemical, nutritional and structural similarities) with long‐standing native plants, compared with novel plants that are more distinct. Additionally, the highly significant positive effect of host plant range size on DBIF richness indicates that it is not only the ‘novelty’ of a novel plant habitat that is important, but also its areal extent. The species‐area effect is well established (Andow & Imura, 1994; Branco et al., 2015; Brändle & Brandl, 2001; Kennedy & Southwood, 1984; Lawton et al., 1993; Strong et al., 1977), but it is rarely considered alongside the effect of phylogenetic isolation. Our results suggest that phylogenetic isolation (expressed as phenotypic divergence) and range size work in tandem to influence the accumulation of richness in novel plant habitats.

It is important to acknowledge that variation in recording effort (measured as log(sources)) in the geographic‐scale DBIF data had the strongest effect on measured species richness, compared to the nonetheless significant effects of host plant phylogenetic isolation, range size and native status. This is a consequence of the large variation in the number of sources reporting data for insects associated with different plant species, combined with the well‐established positive relationship between species richness and recording effort (e.g. Fisher, Corbet, & Williams, 1943). We call for more systematic and controlled sampling to be carried out at these broader geographic‐scales. Despite this ‘noise’ in the DBIF database, the significant effects of phylogenetic isolation at a geographic‐scale are consistent with the conclusions of the tightly controlled, local‐scale experimental plots.

Analyses that considered time since introduction revealed that archaeophytes (pre‐1500 arrivals) were more species‐rich than neophytes (post‐1500 arrivals), indicating species accumulation through time, at least on longer time scales. This is congruent with the conclusions of others in which richness can be observed to increase through time in novel plant and other habitats (both on geological timescales e.g. the last glacial maximum to present day, and successional timescales e.g. a century following forest clearing for agriculture—Brändle et al., 2008; Cramer et al., 2008; Kennedy & Southwood, 1984; Li et al., 2014; Nichols & Nichols, 2003). The specific date of introduction was not significant for the analysis of neophytes in the DBIF data, although the lack of an effect of time on shorter time scales (e.g. Kirichenko & Kenis, 2016) may partly stem from the activity of entomological recorders, which has generally increased over time.

Vortis sampled insects were more specialized than pollinators. These results are consistent with insect pollinators being typically more generalized than other insect herbivores (Fontaine, Thebault, & Dajoz, 2009), and with British pollinators being particularly generalized when compared with pollinators from other regions (Blüthgen et al., 2006). Importantly, the accumulation of the more specialized Vortis insects on host plants was solely influenced by the *nearest* phylogenetic neighbour/native neighbour distance, whereas the more generalized pollinators were influenced both by *mean* phylogenetic isolation/isolation from natives, and *nearest* phylogenetic neighbour/native neighbour distance (richness models only). These results indicate that the characteristics of potential insect colonists may also influence colonization. Specialized insect colonists may be primarily sourced from the single most similar habitat, whereas generalists may be recruited onto novel plant habitats from a wider range of sources.

Several lines of evidence indicate that some nonnative plants play host to a unique and distinctive fauna: some insect species/taxa were uniquely sampled from nonnative plants (9% of pollinators, 32% of Vortis insects and 10% of DBIF insects; Figure 5), exotic plants from the southern hemisphere supported significantly more distinct insect communities (for both pollinator and Vortis samples), phylogenetically isolated nonnative plants supported the most distinctive insect communities (for DBIF data), and the species/taxon richness of insects in landscapes containing a mixture of native and nonnative plants was high (sample‐based accumulation curves). The 9%–32% unique insect taxa on nonnative plants is considerably higher than 1%–3% of insect taxa in the databases that are themselves nonnative species (2% in the DBIF, 3% in the Wisley pollinator samples and 1% in the Wisley Vortis samples; percentages based on the taxa identified to species level and of known historic status), meaning that the distinctive communities on nonnative/phylogenetically isolated plants were primarily formed from the redistribution of rare native insect species, rather than through the establishment of nonnatives in new regions.

We recognize that additional sampling would be beneficial to establish the full host range of every insect in our datasets, but our results appear to contradict the suggestion that nonnative plants solely host a small subset of the assemblages found on native plants (Perre, Loyola, Lewinsohn, & Almeida‐Neto, 2011). This is reminiscent of the way in which several human‐altered habitats (e.g. brownfield sites, mine tailings and green rooves) sometimes contain species that are rare or absent elsewhere (Eyre, Luff, & Woodward, 2003; Jones & Leather, 2012; MacIvor & Lundholm, 2011; Tischew et al., 2014), and thus contribute to regional diversity. While the abundances and taxonomic richness of insects associated with novel plant habitats may be reduced at a local level, novel plant habitats may recruit taxa rarely found in native plant habitats, thus contributing to and potentially maintaining regional diversity. In our local‐scale samples, taxon richness accumulation curves that pooled data for natives, congeners and exotics were not significantly different to the curves for native‐only or congener‐only plants. Similarly, DBIF species richness accumulation curves that pooled data for natives, archaeophytes, and neophytes revealed that mixed landscapes accumulated richness at a similar rate compared with native only long‐standing landscapes. We cannot conclude that the colonization of novel plant habitats by unique taxa will increase overall regional diversity (e.g. Hiley, Bradbury, & Thomas, 2016; Sax & Gaines, 2003; Vellend et al., 2017), but our results imply that a mixture of longer standing and more novel plant habitats may retain diversity, albeit with a changed composition.

Under our framework, we hypothesize that nonnative plants may function as analogues of novel anthropogenic habitats, and that understanding more about the processes underlying insect accumulation on nonnative plants may provide some useful insights into the accumulation of species in novel anthropogenic habitats in general. Whilst we acknowledge that there may be difficulties in mirroring all aspects of novel habitat traits within plant biology, we suggest that there are parallels between introduced plants and other novel habitats. The sometimes divergent structures of nonnative plants (e.g. plant height and branching complexity) and their associated microclimates may relate to the physical diversity of other novel habitats (be they mine tailings or urban heat islands), and nonnative plants also contain an array of chemicals present in their tissues, exudates and associated soils, analogous to the chemical and soil diversity of postindustrial sites. We propose that host plant phylogenetic isolation may capture some of the aforementioned traits with a single metric, by operating as a proxy for the habitat novelty provided by differing host plant phenotypes. Further research is necessary to develop the frameworks required to quantify the relative ‘novelty’ provided by other types of anthropogenic habitats (e.g. comparing green rooves vs. biologically invaded communities), but time since nonnative plant introduction and nonnative plant range size represent the age and area (extent) of a novel habitat. Nonnative plants represent one of the most numerous novel habitat types globally (van Kleunen et al., 2015), and are playing an increasingly prominent ecological role in virtually all landscapes (Schlaepfer et al., 2011). Thus, whilst our model system may not perfectly translate to other novel habitat types, novel plant habitats are certainly abundant, highly replicated and important ecologically, making them an ideal model system to study.

To conclude, the similarity of a novel plant habitat to long‐standing habitats can have a large impact on biological recruitment, affecting the abundances, richness and distinctiveness of the associated biota. Given the influence of phylogenetic position on host plant phenotypic traits (Bezemer et al., 2014; Cappuccino & Arnason, 2006; Rasmann & Agrawal, 2011; Schoonhoven et al., 2005), congeneric nonnatives and species with low phylogenetic isolation were more likely to match natives in a variety of traits that determine how insects consume or otherwise associate with plants. We conclude that:

1. Novel plant habitats that are particularly divergent compared with existing habitats will initially be colonized by fewer taxa and individuals, although these colonists may be particularly distinctive.
2. Novel plant habitats that occupy a larger area will be colonized by more species.
3. Species richness in novel plant habitats will increase over time (plant arrival before vs. after 1500), although the schedule of accumulation on shorter time spans remains uncertain.
4. The ‘novelty’ of a novel plant habitat should be viewed in relation to the attributes of potential colonists. More generalized colonists may respond to the structural, chemical and ecological differences between a novel plant habitat and a wide array of existing habitats, whereas more specialized colonists may be primarily influenced by the differences between the novel plant habitat and a much smaller subset of similar existing habitats.
5. The faunal richness of regions that contain relatively even mixtures of long‐standing and novel plant habitats will be similar to that found in regions with just long‐standing habitats, due to the colonization of novel plant habitats by unusual taxa.

Overall, the more divergent a novel plant habitat is from existing habitats, the lower the total abundance of associated insects and the less local α‐diversity that it will attract (at least initially). However, the higher distinctiveness of its biota may contribute to regional richness.

## CONFLICT OF INTEREST

The authors declare no competing interests.

## Data Availability

Wisley experimental summary and interaction data are available from the Dryad digital repository: https://doi.org/10.5061/dryad.1vhhmgqp4. DBIF summary and interaction data are available from the EIDC: https://doi.org/10.5285/cc6b5e83-a1f4-40d6-bbbb-64366b002418.
