# Supplementary material for: Introduced plants as novel Anthropocene habitats for insects
Source: Glob Chang Biol. 2019 Dec 16;26(2):971–88. doi: 10.1111/gcb.14915 (PMC7027573; doi:10.1111/gcb.14915)
Supplement: Supplementary file 1 [file GCB-26-971-s001.docx]

**Supplementary Information**

Supplementary Text

Pg. 3 – Local-scale plant mixtures

Pg. 3 – Local-scale protocols for horticultural management practice

Pg. 4 – Local-scale pollinator sampling protocol

Pg. 4 – Local-scale Vortis sampling protocol

Pg. 4-5 – Local-scale Vortis plant architecture measurements

Pg. 5 – R packages used for analysis

Pg. 5-6 – Predictor cross correlation analysis

Supplementary Figures

Pg. 7 – Supplementary Figure 1: Local-scale experimental planting plan

Pg. 8 – Supplementary Figure 2: Local-scale experimental plot layout

Pg. 9 – Supplementary Figure 3: Local-scale pollinator NMDS co-ordinates in three dimensional space

Pg. 10 – Supplementary Figure 4: Local-scale Vortis NMDS co-ordinates in three dimensional space

Pg. 11 – Supplementary Figure 5: a) The effect of host plant nearest phylogenetic neighbour distance on local-scale pollinator richness; b) The effect of non-native host plant nearest native phylogenetic neighbour distance on local-scale pollinator richness

Pg. 12 – Supplementary Figure 6: a) The effect of non-native host plant mean phylogenetic isolation from natives on geographic-scale DBIF insect richness; b) The effect of non-native host plant nearest phylogenetic neighbour distance on geographic-scale DBIF insect richness

Pg. 13 – Supplementary Figure 7: Venn diagram displaying the number of local-scale pollinators unique to each host plant native status

Pg. 14 – Supplementary Figure 8: The effect of host plant status on local-scale pollinator community distinctiveness

Pg. 15 – Supplementary Figure 9: The effect of non-native host plant phylogenetic isolation on geographic-scale DBIF insect community distinctiveness

Supplementary Tables

Pg. 16-18 – Supplementary Table 1: Local-scale plant species in each group and native status

Pg. 19 – Supplementary Table 2: Level of identification and primary identification work used for each taxonomic group of local-scale Vortis insects

Pg. 20 – Supplementary Table 3: Likelihood ratio tests determining the significance of the interaction between local-scale Vortis insect feeding type and plant native status, or phylogenetic isolation, when regressed against insect richness and abundance in separate negative binomial models

Pg. 21 – Supplementary Table 4: Negative binomial models describing the effects of host plant native status, and/or host plant phylogenetic isolation, and several control variables (pollinator models only) on local-scale insect abundance

Pg. 22 – Supplementary Table 5: Negative binomial models describing the effects of host plant native status, and/or host plant phylogenetic isolation, and several control variables (pollinator models only) on local-scale insect richness

Pg. 23 – Supplementary Table 6: Poisson/negative binomial models describing the effects of host plant native status, neophyte host plant arrival date, host plant phylogenetic isolation, and host plant range size (no. of hectads) on geographic-scale DBIF insect richness.

Pg. 24 – Supplementary Table 7: Quasi-binomial models describing the effects of host plant native status, and/or host plant phylogenetic isolation, and pollinator host plant replicate number on local-scale insect community distinctiveness

Pg. 25 – Supplementary Table 8: Quasi-binomial models describing the effects of host plant native status, neophyte host plant arrival date , host plant phylogenetic isolation, and host plant range size (no. of hectads) on DBIF insect community distinctiveness.

References

Pg. 26-29

**Local-scale plant mixtures**

Each mixture of plants contained 14 species, as detailed in **Supplementary Table 1**, and each plant species was planted in a standardized position within its plot (**Supplementary Fig. 1**). The positions of the plots on the two experimental sites are shown in **Supplementary Fig. 2**.

**Local-scale protocols for horticultural management practice**

Initial planting took place between May 2009 and June 2010, and subsequent management followed the protocols of Salisbury et al*.* (2015). In summary:

Weed control: All plants not intentionally planted were hand weeded to prevent competition with the plant assemblages and the provision of resources that could potentially be used by insects. Self-sown seedlings of plants within the planting scheme were also removed unless required to fill gaps of that plant within its allotted space.

Pesticides: No pesticides were used.

Plant restriction, support and pruning: Plants were allowed to flourish within the area defined by the planting plan (**Figure S1**). Any plant material that overhung the edge of a plot to the extent that it touched the ground, reached a height above 2.4 m or had encroached beyond its allotted space within a plot was restricted, by pruning, staking or removal. Pruning to restrict growth was carried out in such a way as to minimize loss of flowers and/or seed-heads. Plants were not deadheaded (‘removal of dead and dying flowers’) as any seed/fruit set would be lost with potential effects on insects associated with that resource. Dead stems of herbaceous perennials were left standing through winter and cut back in February, conforming to ‘wildlife-friendly’ gardening advice (Baines, 2000).

Irrigation**:** Watering was carried out as required to enable plant establishment, and to ensure plant survival during drought conditions (e.g. summer 2013).

Winter protection: From 2012/13 onwards, a dry mulch of straw was applied to protect a number of plants that had been lost in preceding winters.

Plant replacement: Occasional plant failures (e.g. because of winter losses, disease, or turnover in short-lived perennials) were normally replaced by the same species (and cultivar). In some instances, alternative plants cultivars/species were used, as detailed in **Supplementary Table 1**.

**Local-scale pollinator sampling protocol**

Recording occurred between May and September when temperatures were greater than 17 °C, wind speeds were less than 5 on the Beaufort scale, and it was neither raining nor likely to rain. In March and April recording took place when temperatures were greater than 8 °C, it was not raining, cloud cover was less than 25%, and wind speed was less than 2 on the Beaufort scale. To minimise the effects of possible diurnal variation in insect visits, each plot was visited twice on each sampling day. Morning sampling sessions started between 09:00 and 10:00, and afternoon sampling sessions between 13:00 and 14:00. Consequently, each plot was sampled for eight minutes on each sampling day. The two experimental sites were visited on different days, with sampling of the second site always occurring within seven days of the first. The order of visiting each plot type (plant mixture) was randomised prior to each recording event.

**Local-scale Vortis sampling protocol**

Vortis sampling occurred after 10:00, when vegetation was dry to the touch, and with temperatures greater than 17°C. The two experimental sites were sampled alternately, with sampling sessions rotating between them. Vortis sampling was carried out by sweeping the suction nozzle across half of each individual plant for 30 seconds. From ground level the Vortis was moved in a sweeping motion up the plant (ensuring not to touch the ground to avoid accidental sampling of soil invertebrates), terminating at the top of the plant, or otherwise at a height of 1.5 metres. To provide consistency, R. Padovani carried out all Vortis sampling. After sampling each plant, the collection tube was removed, stored in a cool box in the field, then sorted using a pooter in the laboratory to separate insects from plant debris (after being stored at 4-10 °C for ~20 minutes to reduce insect activity). Insects were then frozen and stored at -20 °C until identification.

**Local-scale Vortis plant architecture methods**

Plant Area: Overhead photographs (Sony Cyber-shot DSC- HX1 camera) were taken of each plot from a 3.6 m tripod ladder (Niwaki, Somerset, UK), with a 1 m rule placed in shot as a reference. If a plant was obscured from the overhead perspective by another plant, photographs were taken from the side of each bed, whilst holding the camera as high as possible over the plant in question, in order to retain a near-vertical position over the plant. The area of each plant was then measured using the program ImageJ (Schneider, Rasband, & Eliceiri, 2012). Some plants were obscured from overhead photographs and too tall for manual photographs. In these cases, estimates of area were taken through direct measurement with a rule of the length and width of each plant along two perpendicular axes. The height of each plant was multiplied by its area, to give an overall index of volume for inclusion in the analyses.

Branching Architecture: The branching architecture (apical dominance index) of each plant species was taken to be the number of (living/with leaves) divisions along each branch, divided by the length of branch. The length of a branch was the distance (cm) from the tip of a terminal, leaf-bearing branch, down the stem until we encountered a dead (leafless) sub-branch. This was measured for three branches on the median height individual of each plant species, and the average taken.

**R packages used for analysis**

A list of R packages used for various functions is as follows: data manipulation = reshape (Wickham, 2007), plyr (Wickham, 2011), stringr (Wickham, 2017), and data.table (Dowle & Srinivasan, 2017); Chao-Sorensen dissimilarity index = CommEcol (Melo, 2017); NMDS analysis = vegan (Oksanen et al*.*, 2018); d’ specialisation index calculation = bipartite (Dormann, Gruber, & Fruend, 2008); sample based rarefaction = iNEXT (Hsieh, Ma, & Chao, 2016); D² calculation = modEvA (Barbosa, Brown, Jimenez-Valverde, & Real, 2016); likelihood ratio tests = lmtest (Zeileis & Hothorn, 2002); negative binomial models = MASS (Venables & Ripley, 2002); beta models = betreg (Cribari-Neto & Zeileis, 2010); type II deviance calculation = car (Fox & Weisberg, 2019); post-hoc Tukey contrasts = multcomp (Hothorn, Bretz, & Westfall, 2008); figures = ggplot2 (Wickham, 2009); adding significance bars to box plots = ggsignif (Ahlmann-Eltze, 2017); beta regression figures = sjPlot (Lüdecke, 2019); Venn diagrams = limma (Ritchie et al*.*, 2015).

**Predictor cross-correlation analysis**

We examined correlations among all predictor variables which were candidates for inclusion in models.

Local-scale: The effect of host plant native status (native, congeneric non-native, exotic non-native) on all other pollinator and Vortis model predictors was examined using Kruskal-Wallis tests. The only significant effect of status (other than those shown in the main results) was on pollinator host plant median Julian date (χ2 = 9.21, p = 0.010, d.f. = 2). However, this predictor did not significantly improve the overall pollinator models, and so was not included in the final analysis. All other continuous pollinator and Vortis model predictors were tested for correlation with each other using Kendall Tau-b correlation. No significant correlations existed between Vortis predictors included in the models for final analysis. There were several correlations between pollinator model phylogenetic predictors (mean phylogenetic isolation, nearest phylogenetic neighbour distance, and non-native host plant mean phylogenetic isolation from natives) and the number of host plant replicates, but these were weak (tau = -0.238, -0.229, -0.244), and were therefore unlikely to have impacted our analyses.

Geographic-scale: Host plant native status (native, archaeophyte, neophyte) was significantly or marginally (0.05 < p < 0.1) associated with all DBIF model predictors (i.e. sources Kruskal-Wallis χ2 = 68.70, p < 1e-04, d.f. = 2; hectads χ2 = 185.00, p < 1e-04, d.f. = 2; mean phylogenetic isolation χ2 = 8.76, p = 0.013, d.f. = 2; nearest phylogenetic neighbour distance χ2 = 26.81, p < 1e-04, d.f. = 2; non-native host plant mean phylogenetic isolation from natives χ2 = 3.70, p = 0.054, d.f. = 1; non-native host plant nearest native phylogenetic neighbour distance χ2 = 30.74, p < 1e-04, d.f. = 1). This may explain the loss of significance of two of our four metrics of phylogenetic isolation when included in the same model as host plant native status (**Supplementary Table 6**).

Sampling effort (the number of separate sources in the DBIF database reporting insects associated with a particular plant species) was correlated with non-native host plant mean phylogenetic isolation from natives, host plant range size (hectads) and neophyte introduction date, however the correlations were weak (Kendall Tau-b correlation tau = 0.08, 0.38 and 0.14, respectively), and were therefore unlikely to have impacted our analyses. Host plant range size (hectads) and neophyte introduction date were also weakly correlated (tau = 0.23).


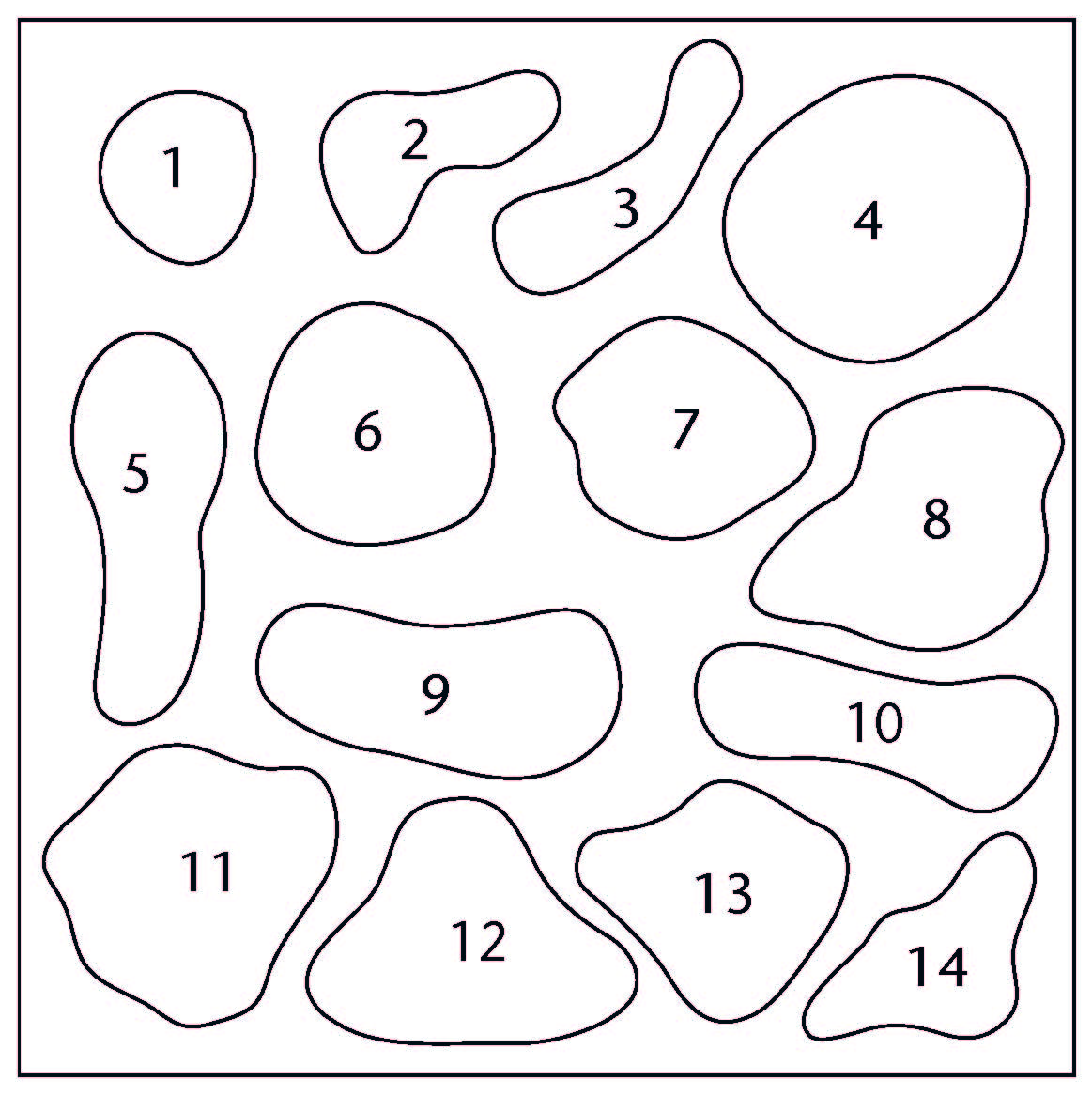


**Supplementary Figure 1.** Local-scale experimental planting plan (Adapted from figure previously published in supplementary information with Salisbury et al*.* 2015). The layout of plant species within each 3 x 3m plot followed a standardized pattern. Within each plot the plant species labels represent the following: 1, Climber; 2, Perennial (deciduous or groundcover); 3, Perennial (deciduous or bulbous); 4, Perennial (deciduous); 5, Perennial (grass/grass-like plant or fern); 6, Shrub; 7, Shrub; 8, Shrub or perennial (deciduous); 9, Shrub or perennial (deciduous); 10, Low growing shrub or perennial (deciduous); 11, Perennial (deciduous); 12, Perennial (evergreen); 13, Perennial (deciduous); 14, Low shrub or perennial (deciduous). Note that the term ‘perennial’ is used here in its horticultural meaning as a noun (i.e. a non-woody plant that lives for multiple years) not an adjective (i.e. a descriptive term for any plant that lives for multiple years).


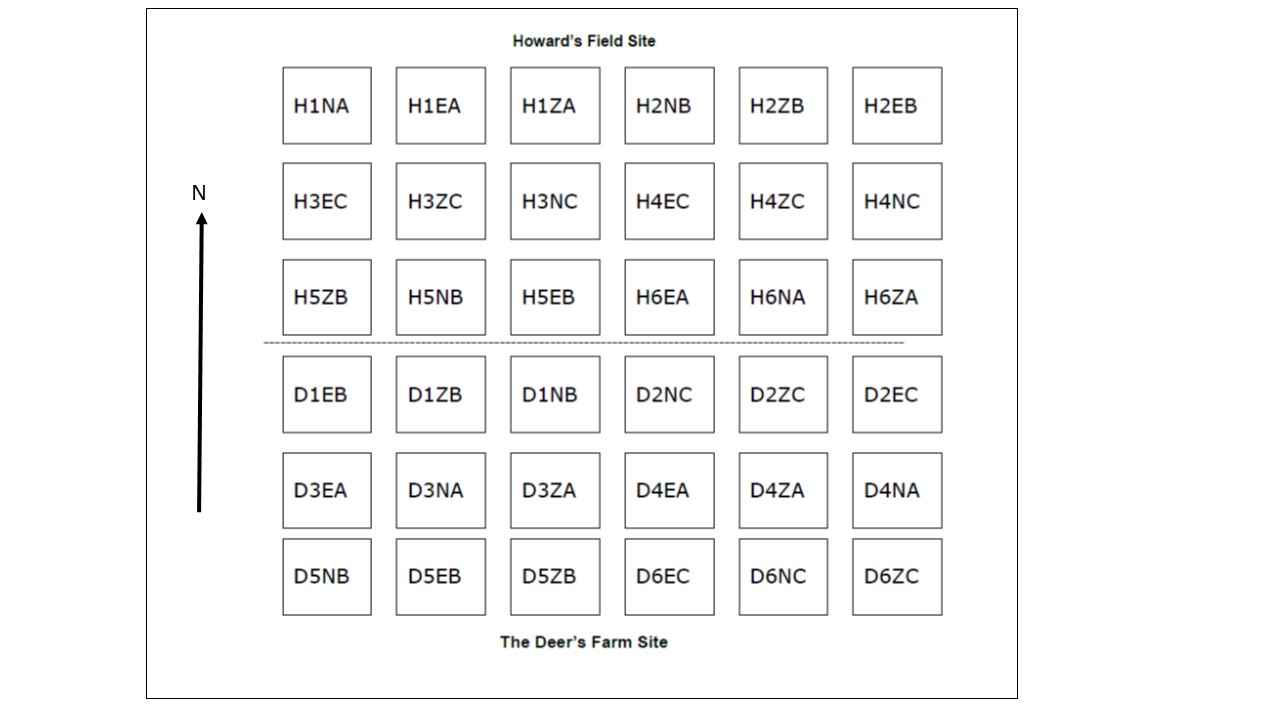


**Supplementary Figure 2:** Local-scale experimental plot layout (Adapted from figure previously published in supplementary information with Salisbury et al*.* 2017). H = Site 1 (Howard’s Field), D= Site 2 (Deer’s Farm), 1 – 6 represent triplet organisation at each site, N = Native Plants, Z = Congener Plants, E = Exotic Plants, A, B, C = Plant Group (See Table S1)

**
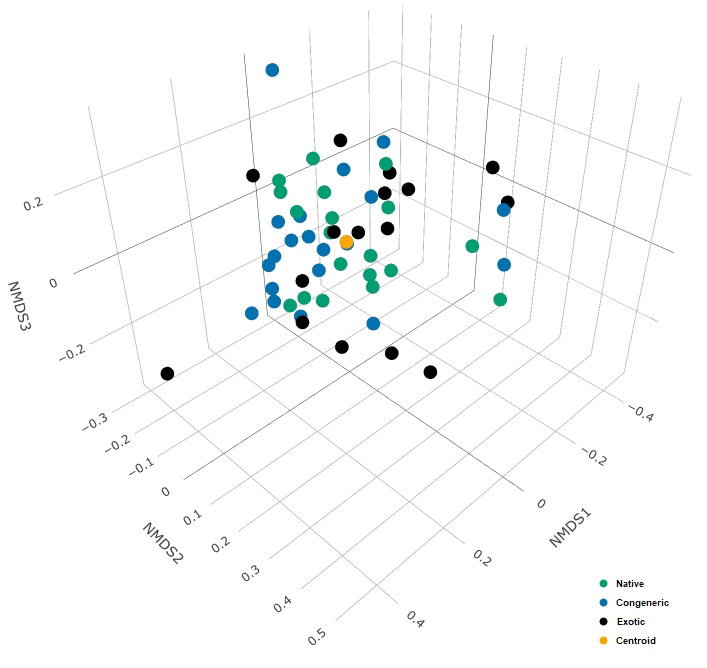
**

**Supplementary Figure 3:** Three-dimensional non-metric multidimensional scaling (NMDS) of Chao-Sorensen abundance-based dissimilarities of local-scale pollinator communities. Stress = 0.165, indicating a good representation of the data in the reduced dimensions. Each point represents a plant species. The distance from each point to the group centroid (0, 0, 0) represents the distinctiveness of the insect community on that plant species.

**
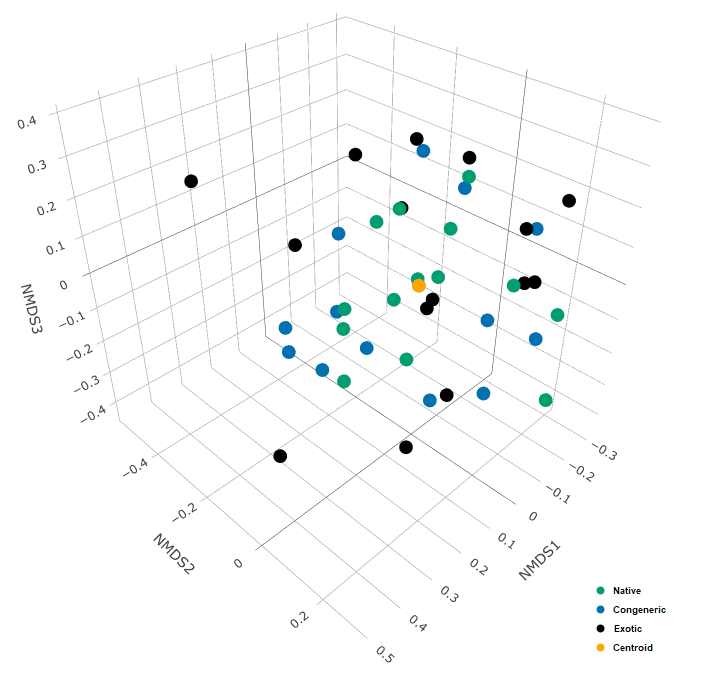
**

**Supplementary Figure 4:** Three-dimensional non-metric multidimensional scaling (NMDS) of Chao-Sorensen abundance-based dissimilarities of local-scale Vortis insect communities. Stress = 0.161, indicating a good representation of the data in the reduced dimensions. Each point represents a plant species. The distance from each point to the group centroid (0, 0, 0) represents the distinctiveness of the insect community on that plant species.

**
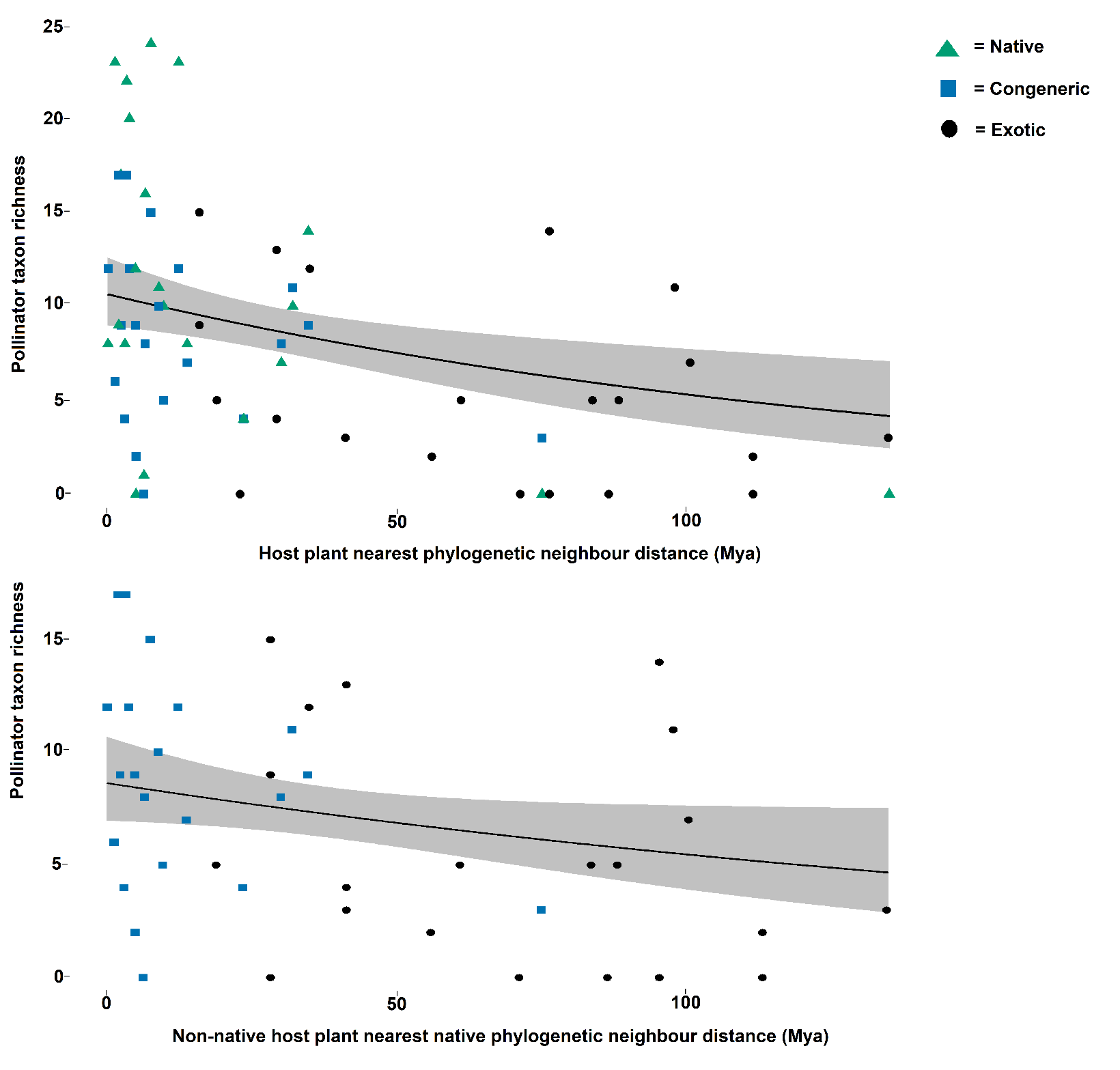

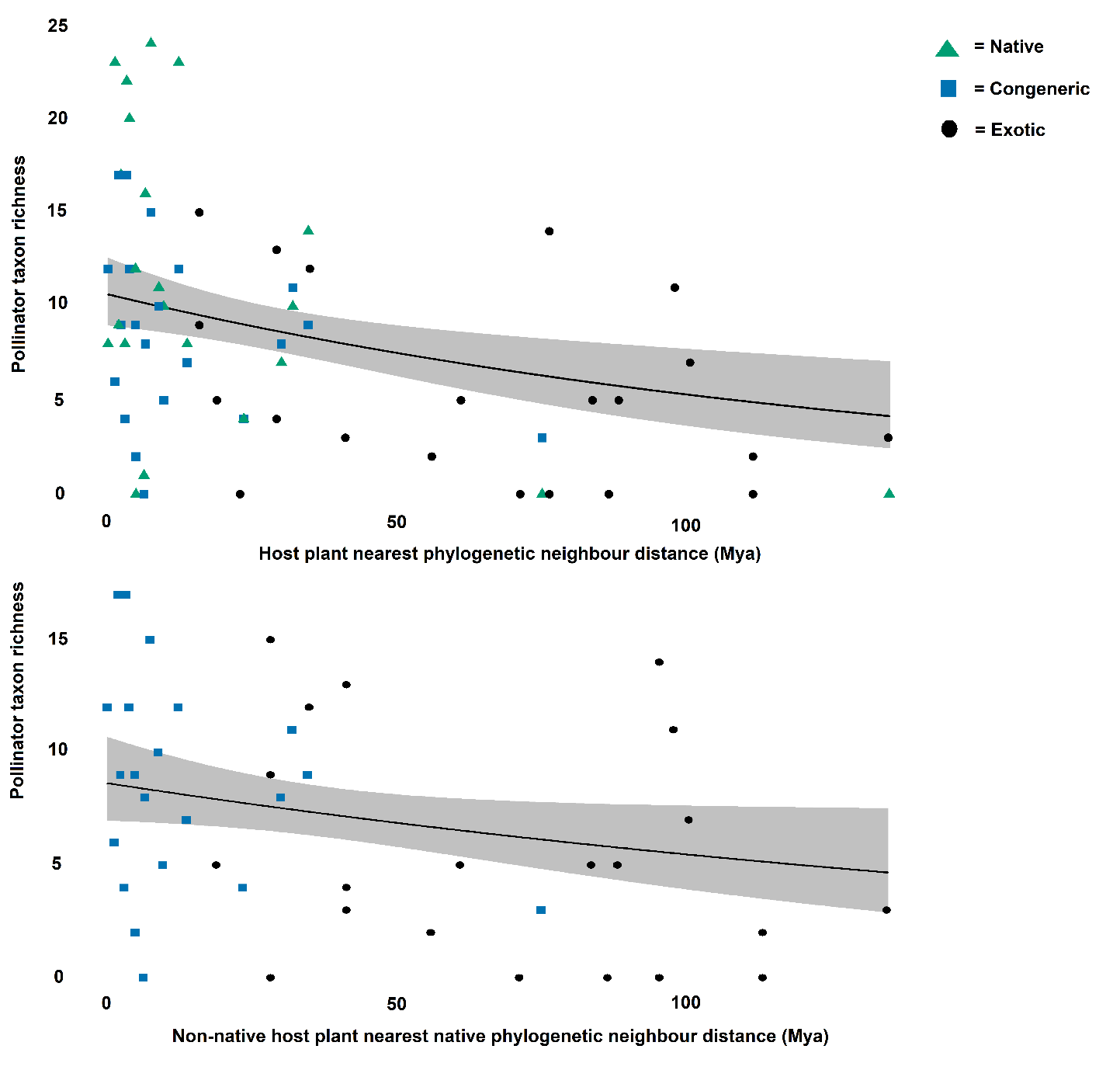
**

**Supplementary Figure 5:** The effect of host plant phylogenetic isolation on local-scale pollinator abundance and taxon richness. Partial regression plots display the effect of our focal predictor (phylogenetic isolation), whilst holding all other predictors at their mean. Shaded areas represent 95% confidence intervals. Data points represent individual plants species. Nearest phylogenetic neighbour distance (NPN) = distance in millions of years from host plant to closest phylogenetic neighbour in the local community. Non-native host plant nearest native phylogenetic neighbour distance (NPNN) = distance in millions of years from *non-native* host plant to closest *native* phylogenetic neighbour in the local community. See Methods for details of the calculation of D² and D.

**a)** Negative binomial model (Pollinator Taxon Richness ~ log(Replicates) + Flowering Units + NPN), n = 64, p(NPN) = 0.003, D² = 0.611, D = 0.120 – 0.389.

**b)** Negative binomial model (Pollinator Taxon Richness ~ log(Replicates) + Flowering Units + NPNN), n = 42**,** p(NPNN) = 0.039, D² = 0.628, D = 0.121 – 0.311.

**
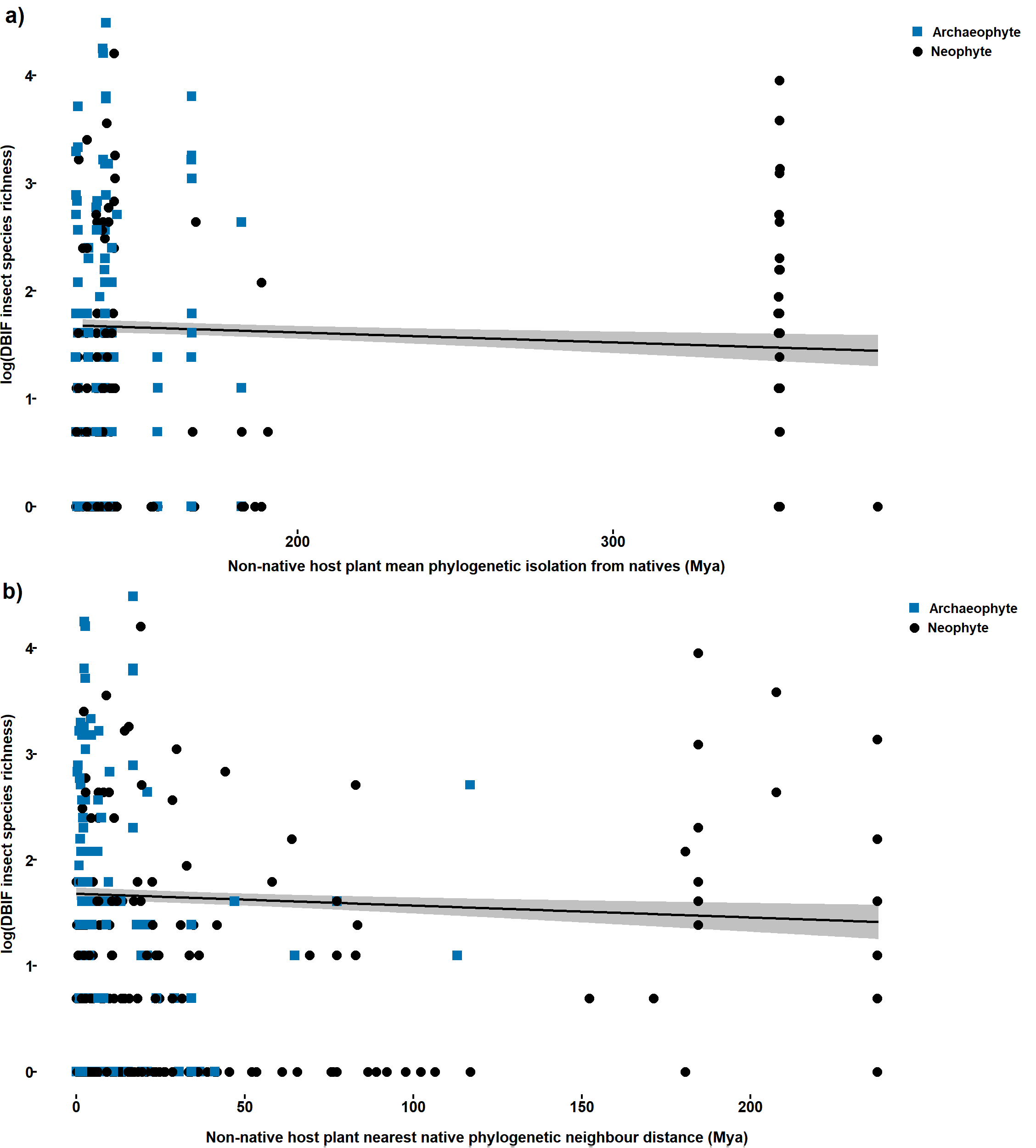
**

**Supplementary Figure 6:** The effect of phylogenetic isolation on geographic-scale DBIF insect species richness. Partial regression plots display the effect of our focal predictor (phylogenetic isolation), whilst holding all other predictors at their mean. Shaded areas represent 95% confidence intervals. Data points represent individual plants species. Non-native host plant mean phylogenetic isolation from natives (PIN) = mean distance in millions of years from *non-native* host plant to all other *native* plants in the DBIF. Non-native host plant nearest native phylogenetic neighbour distance (NPNN) = distance in millions of years from *non-native* host plant to closest *native* phylogenetic neighbour in the DBIF. See Methods for details of the calculation of D² and D.

**a)** Poisson model (Richness ~ log(Sources) + PIN + Hectads) n = 352, p (PIN) = 0.003, D² = 0.924, D = 0.014 – 0.769.

**b)** Poisson model (Richness ~ log(Sources) + NPNN + Hectads) n = 352, p (NPNN) = 0.002, D² = 0.924, D = 0.016 – 0.758.


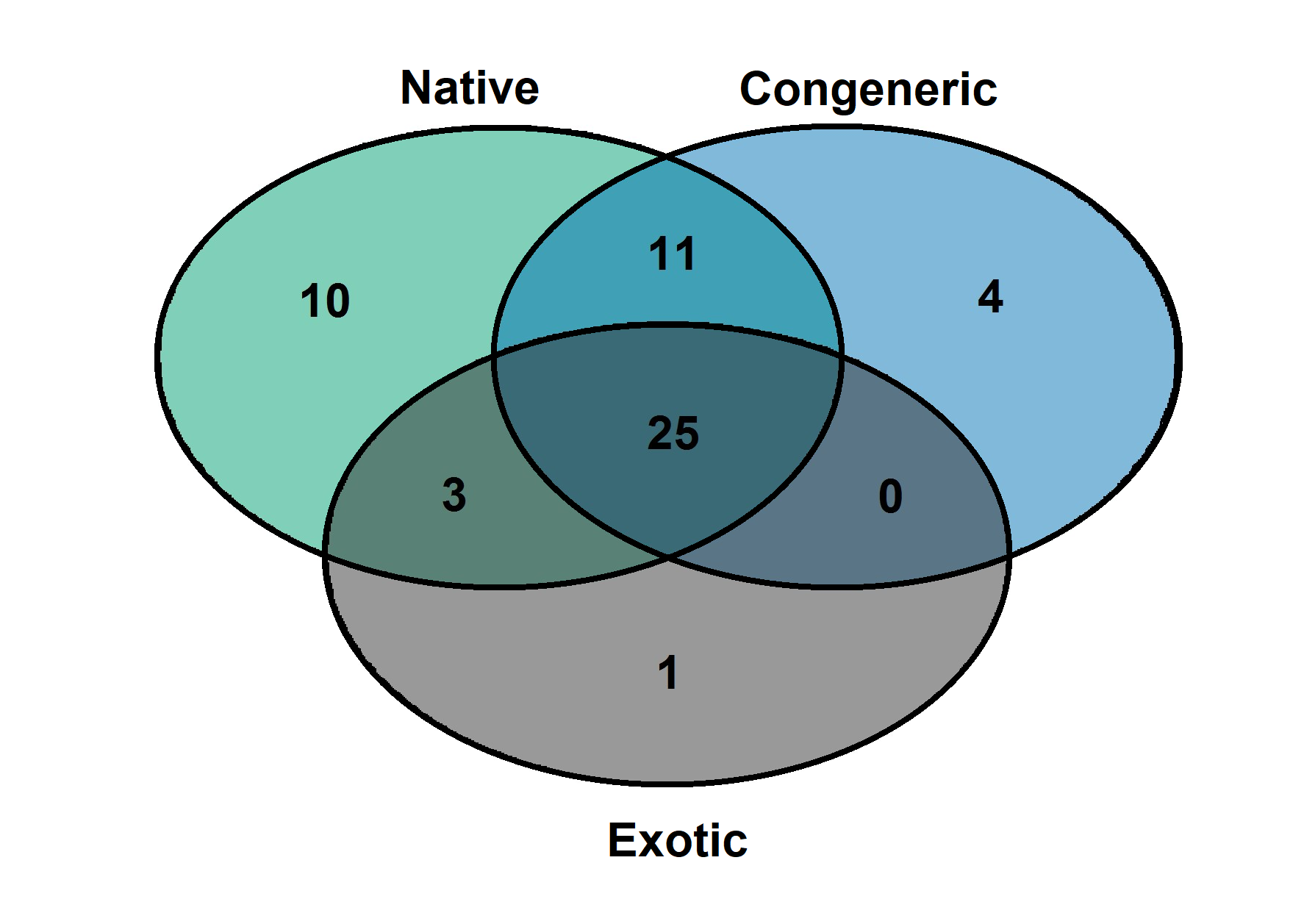


**Supplementary Figure 7:** Venn diagram displaying the number of local-scale pollinators unique to, and shared between each host plant native status. Sample size of Native = 23 plant species, Congeneric = 21, Exotic = 20.

**
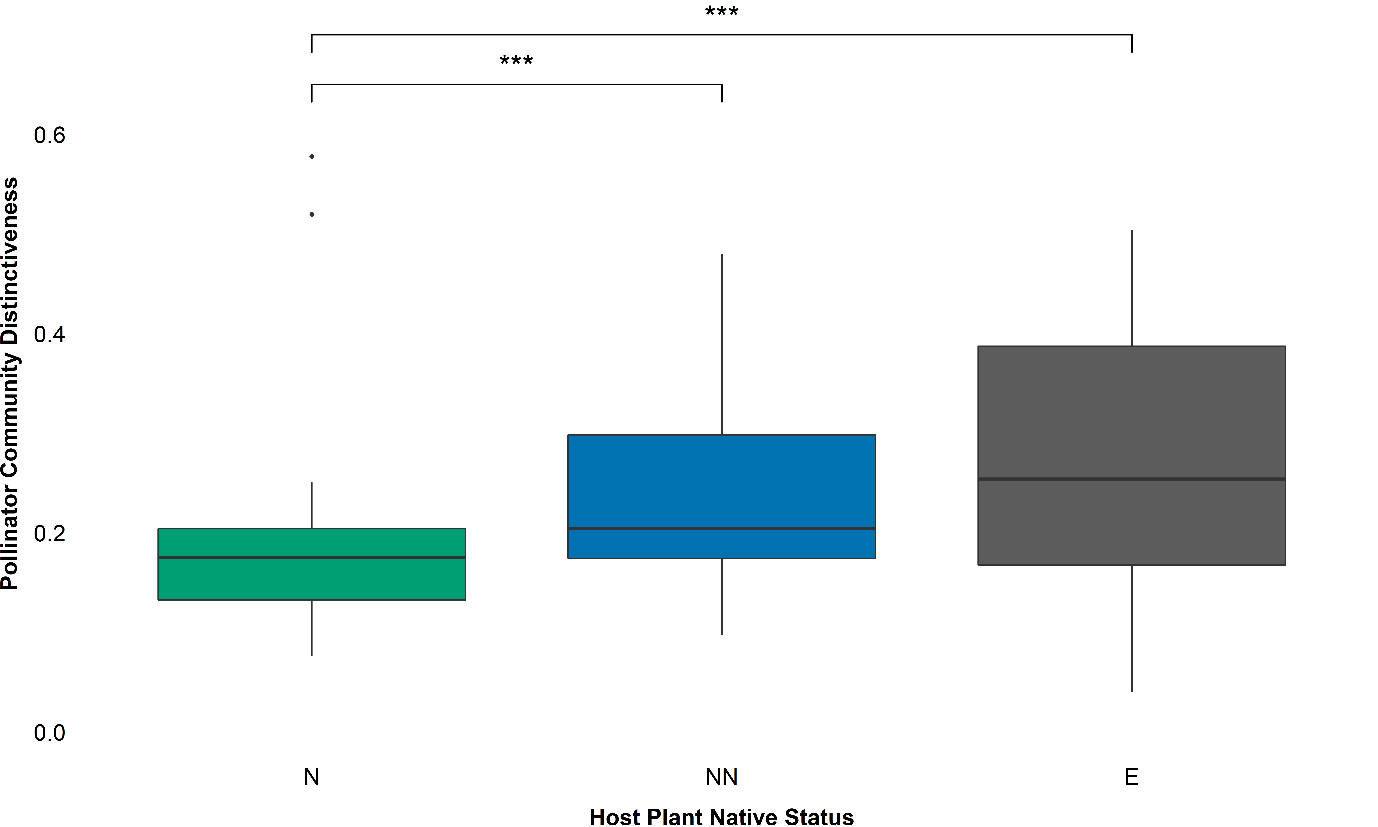
**

**Supplementary Figure 8:** Local-scale pollinator insect community distinctiveness on the different host plant statuses. Pollinator insect distinctiveness was calculated using a non-metric multidimensional scaling approach (see Methods). Boxplots represent median, interquartile range, and 1.5x the interquartile range. Boxplot points represent outliers. *** = significance of Tukey post-hoc contrasts < 0.001. Beta model (Pollinator Insect Community Distinctiveness ~ log(Replicates) + Flowering Units + Status | Status + Median Julian Sampling Date) pseudo R² = 0.235. Sample size of Native = 19 plant species, Congeneric = 20, Exotic = 16. See Methods for the distinction between beta regression mean and precision submodels.

**
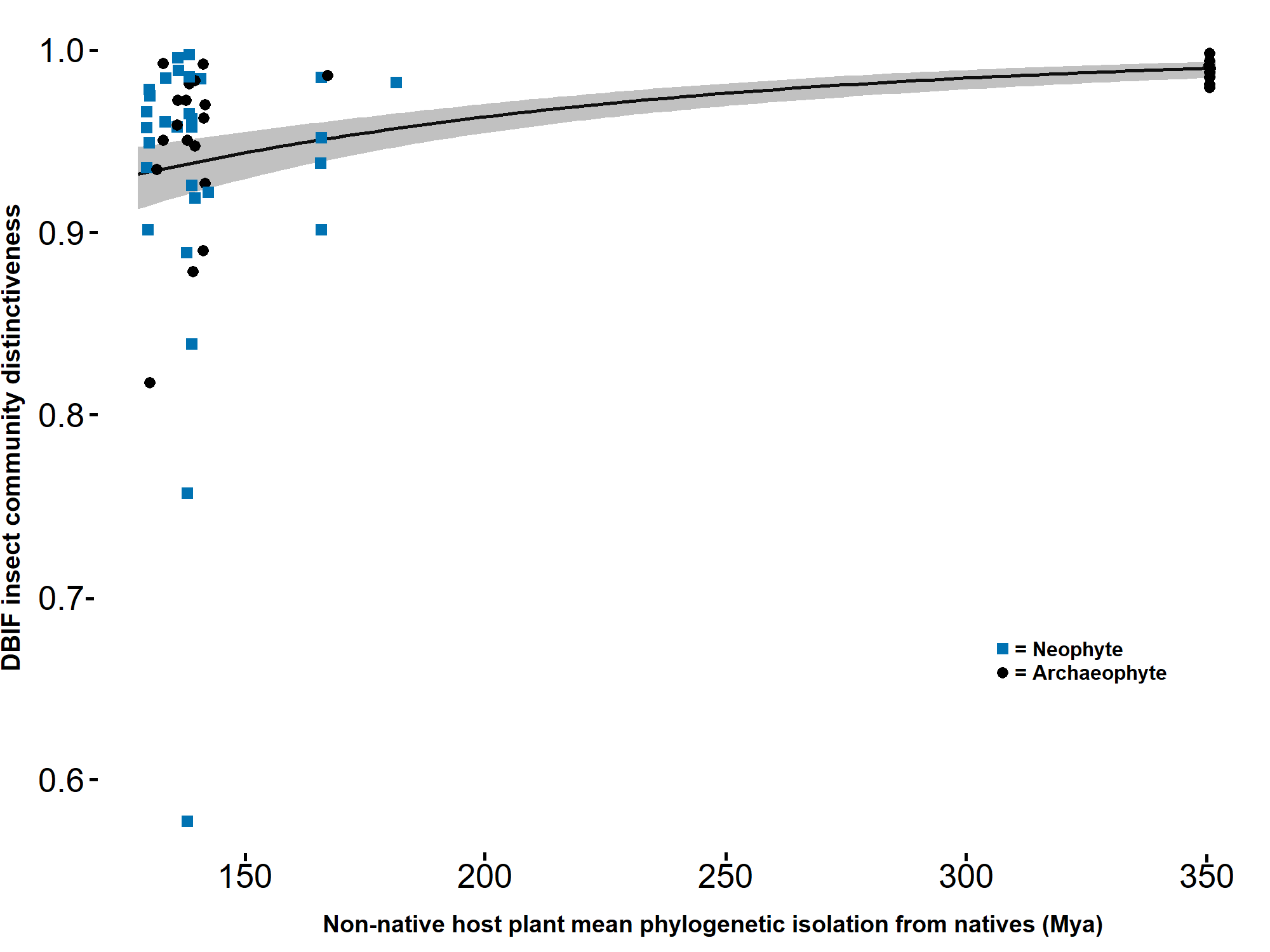
**

**Supplementary Figure 9:** The effect of non-native host plant phylogenetic isolation on geographic-scale DBIF insect community distinctiveness. A partial regression plot displays the effect of our focal predictor (phylogenetic isolation), whilst holding all other predictors at their mean. Shaded areas represent 95% confidence intervals. Data points represent individual plants species. Non-native host plant mean phylogenetic isolation from natives (PIN) = mean distance in millions of years from *non-native* host plant to all other *native* plants in the DBIF. The distinctiveness of the insect community on a plant was represented by dissimilarity from the pool of insects found on native plants (see Methods). Beta model (DBIF Insect Community Distinctiveness ~ log(Sources) + PIN | PIN) n = 56, p(PIN) < 1e-04, pseudo R² = 0.277. See Methods for details of the calculation of pseudo R² and for the distinction between beta regression mean and precision submodels.

**Supplementary Table 1.** Plant species in each species group and native status on the local-scale plots (Adapted from table previously published in supplementary information with Salisbury et al*.* 2015). Not all plant species were included in each analysis. Criteria for inclusion are detailed in main text. For a full list of all plant species with associated plant traits and insect diversity see raw data in *Wisley Experimental Data.xlsx*. In three cases exotic plants were part of the same family as the native plant in their species triplet. These were *Euryops tysonii* (Asteraceae - grouped with *Leucanthemum vulgare*), *Hebe rakaiensis* (Plantaginaceae – grouped with *Veronica spicata*), and Polystichum proliferum (Dryopteridaceae – grouped with *Dryopteris filix-mas*). In all other cases exotics were more distantly related. N = native, C = congeneric, E = exotic. The species mixtures referred to in the main text consist of the 14 native species in group A (the first mixture), the 14 congeneric species in group A (the second mixture), and so on, through to the 14 exotic species in group C (the ninth mixture). The numbers (first column) refer to the position that each plant species was planted in each plot (Supplementary Figure 1) of a given mixture type, and each row in the table connects a given species triplet.

| **Position in Plot** | **Native** | **Congeneric** | **Exotic** | **Triplet Included in Vortis Analysis** | **Triplet Included in Pollinator Analysis** |
| --- | --- | --- | --- | --- | --- |
| **Species group A** | | | | | |
| 1 | *Lonicera periclymenum* 'Graham Thomas' | *Lonicera tragophylla* *1* | *Eccremocarpus scaber* | Just N and C | Yes |
| 2 | *Primula vulgaris* | *Primula japonica* 'Miller's Crimson' | *Oxalis adenophylla* | No | Yes |
| 3 | *Hyacinthoides non-scripta* | *Hyacinthoides hispanica* *2* | *Nerine bowdenii or Ornithogalum candicans* *3* | Just E | Yes |
| 4 | *Valeriana officinalis* | *Valeriana phu* 'Aurea' | *Diascia personata* 'Hopleys'*4* | Yes | Yes |
| 5 | *Deschampsia cespitosa* | *Stipa tenuissima* | *Uncinia rubra* Colenso *6* | Yes | No |
| 6 | *Buxus sempervirens* | *Sarcococca hookeriana* var. *humilis* | *Pittosporum tenuifolium* | Yes | Yes |
| 7 | *Viburnum opulus* | *Viburnum sargentii* | *Ozothamnus rosmarinifolius or Azara serrata* *5* | Just E | Yes |
| 8 | *Lythrum salicaria* | *Lythrum virgatum*  'Dropmore Purple' | *Mirabilis jalapa* *4* | Yes | Yes |
| 9 | *Cytisus scoparius* | *Genista lydia* Boiss. | *Callistemon rigidus** | No | Yes |
| 10 | *Geranium sanguineum* | *Geranium macrorrhizum* | *Leptinella squalida* 'Platt's Black' | Yes | Yes |
| 11 | *Stachys officinalis* | *Stachys byzantina* | *Lobelia tupa* *4* | Yes | Yes |
| 12 | *Armeria maritima* | *Armeria juniperifolia* | *Sisyrinchium striatum* Sm. | Yes | Yes |
| 13 | *Scabiosa columbaria* | *Scabiosa caucasica* | *Eryngium agavifolium* | No | Yes |
| 14 | *Leucanthemum vulgare* (Vail) | *Rhodanthemum hosmariense or Anthemis punctata* *7* | *Euryops pectinatus* or *Euryops tysonii* *6*,*8* | Yes | Yes |
| **Species group B** | | | | | |
| 1 | *Lonicera periclymenum* 'Graham Thomas' | *Lonicera tragophylla* *1* | *Eccremocarpus scaber* | Just N and C | Yes |
| 2 | *Dianthus deltoides* | *Dianthus plumarius* | *Acaena microphylla* | Yes | Yes |
| 3 | *Primula vulgaris* | *Primula japonica* 'Miller's Crimson' | *Oxalis adenophylla* | No | Yes |
| 4 | *Eupatorium cannabinum* | *Eupatorium maculatum* 'Orchard Dene' | *Verbena bonariensis* | No | Yes |
| 5 | *Dryopteris filix-mas*  Schott | *Dryopteris wallichiana* | *Polystichum proliferum* | No | No |
| 6 | *Buxus sempervirens* | *Sarcococca hookeriana* var. *humilis* | *Pittosporum tenuifolium* | Yes | Yes |
| 7 | *Rosa rubiginosa* | *Rosa glauca* | *Fuchsia magellanica* var. *gracilis* | Just N and E | Yes |
| 8 | *Lythrum salicaria* | *Lythrum virgatum* 'Dropmore Purple' | *Mirabilis jalapa* *4* | Yes | Yes |
| 9 | *Cytisus scoparius* | *Genista lydia* | *Callistemon rigidus** | No | Yes |
| 10 | *Geranium sanguineum* | *Geranium macrorrhizum* | *Leptinella squalida* 'Platt's Black' | Yes | Yes |
| 11 | *Knautia arvensis* | *Knautia macedonica* | *Alstroemeria psittacina* | Yes | Yes |
| 12 | *Armeria maritima* | *Armeria juniperifolia* | *Sisyrinchium striatum* | Yes | Yes |
| 13 | *Malva moschata or Silene uniflora* *9* | *Malva alcea or Lychnis flos-jovis* *10* | *Osteospermum jucundum* | Yes | Yes |
| 14 | *Leucanthemum vulgare* | *Rhodanthemum hosmariense or Anthemis punctata* *7* | *Euryops pectinatus* or *Euryops tysonii* *6*,*8* | Yes | Yes |
| **Species group C** | | | | | |
| 1 | *Lonicera periclymenum* 'Graham Thomas' | *Lonicera tragophylla* *1* | *Eccremocarpus scaber* | Just N and C | Yes |
| 2 | *Dianthus deltoides* | *Dianthus plumarius* | *Acaena microphylla* | Yes | Yes |
| 3 | *Hyacinthoides non-scripta* *2* | *Hyacinthoides hispanica* *2* | *Nerine bowdenii or Ornithogalum candicans* *3* | Just E | Yes |
| 4 | *Eupatorium cannabinum* | *Eupatorium maculatum* 'Orchard Dene' | *Verbena bonariensis* | No | Yes |
| 5 | *Molinia caerulea*  Moench | *Calamagrostis brachytricha* | *Carex testacea* | Yes | Just N |
| 6 | *Buxus sempervirens* | *Sarcococca hookeriana* var. *humilis* | *Pittosporum tenuifolium* | Yes | Yes |
| 7 | *Rosa rubiginosa* | *Rosa glauca* | *Fuchsia magellanica* var. *gracilis* | Just N and E | Yes |
| 8 | *Veronica spicata* | *Veronica austriaca*  subsp. *teucrium* | *Hebe rakaiensis* | No | Yes |
| 9 | *Malva moschata or Silene uniflora* *9* | *Malva alcea or Lychnis flos-jovis* *10* | *Osteospermum jucundum* | Yes | Yes |
| 10 | *Helianthemum nummularium* | *Halimium umbellatum* | *Brachyglottis monroi* | No | Yes |
| 11 | *Stachys officinalis* | *Stachys byzantina* | *Lobelia tupa* *4* | Yes | Yes |
| 12 | *Armeria maritima* | *Armeria juniperifolia* | *Sisyrinchium striatum* | Yes | Yes |
| 13 | *Scabiosa columbaria* | *Scabiosa caucasica* | *Eryngium agavifolium* | No | Yes |
| 14 | *Leucanthemum vulgare* | *Rhodanthemum hosmariense or Anthemis punctata* *7* | *Euryops pectinatus* or *Euryops tysonii* *6*,*8* | Yes | Yes |
| *1* Incorrect species supplied – replaced with correct species spring 2011  *2* Incorrect species supplied – replaced with correct species in summer 2010  *3* Original *Nerine bowdenii* (pollinator sampling) replaced by *Ornithogalum candicans* in spring 2015 (Vortis sampling)  *4* Plants replaced by the same species due to winter losses in 2010/11  *5* Original *Ozothamnus rosmarinifolius* (pollinator sampling) died due to *Phytophora* root rot – replaced spring 2011, by *Azara serrata* (pollinator and Vortis sampling)  *6* Plants replaced by the same species due to winter losses in 2011/12  *7* Original *Rhodanthemum hosmariense* (pollinator sampling) replaced by *Anthemis punctata* in spring 2015 (Vortis sampling)  *8* Replaced *Euryops pectinatus* (pollinator sampling), which was lost in winter 2010/11, by *Euryops tysonii* (pollinator and Vortis sampling)  *9* Original *Malva moschata* (pollinators) replaced in spring 2015 by *Silene uniflora* (Vortis sampling)  *10* Original *Malva alcea* (pollinators) replaced by *Lychnis flos-jovis* in spring 2015 (Vortis sampling) | | | | | |

**Supplementary Table 2.** Insect identification of local-scale Vortis samples for allocation of functional group.

| **Taxonomic group** | **Level of Identification** | **Primary identification work** |
| --- | --- | --- |
| HEMIPTERA | | |
| STERNORRHYNCHA | Suborder/Species | Hodkinson & White, 1979; Unwin, 2001 |
| AUCHENORHYNCHA | Genus/Species | Le Quesne, 1965; Le Quesne & Payne, 1981 |
| HETEROPTERA | Genus/Species | Southwood & Leston, 1959 |
| COLEOPTERA | | |
| Anthicidae | Species | Buck, 1954 |
| Apionidae | Species | Morris, 1990 |
| Carabidae | Species | Luff, 2007 |
| Chrysomelidae | Genus/Species | Hubble, 2010 |
| Coccinellidae | Species | Majerus & Kearns, 1989 |
| Corylophidae | Family | Unwin, 1984 |
| Curculionidae | Genus/Species | Joy, 1932; Duffy, 1953; Morris, 1990, 1997, 2008 |
| Lathridiidae | Species | Hackston, 2018 |
| Nanophydiae | Species | Morris, 1990 |
| Nitidulidae | Species | Kirk-Spriggs, 1996 |
| Oedemeridae | Species | Buck, 1954 |
| Ptiliidae | Family | Unwin, 1984 |
| Scraptiidae | Species | Levey, 2009 |
| Staphylinidae | Family/Genus/Species | Joy, 1932 |
| Tenebrionidae | Species | Buck, 1954 |
| Throscidae | Species | Joy, 1932 |
| OTHER ORDERS | | |
| BLATTODEA | Species | Barnard, 2011 |
| DERMAPTERA | Species | Hincks, 1949 |
| ORTHOPTEROID | Species/Genus | Marshall & Haes, 1988 |

**Supplementary Table 3:** Likelihood ratio tests determining the significance of the interaction between local-scale Vortis insect feeding type and plant native status, or phylogenetic isolation, when regressed against insect richness and abundance in separate negative binomial models.

| **Response** | **Predictors without Interaction** | **Predictors with Interaction** | **χ2** | ***p*** | ***d.f.*** |
| --- | --- | --- | --- | --- | --- |
| Abundance | Type + Status | Type*Status | 8.641 | 0.195 | 6 |
| Abundance | Type + Mean PI | Type*Mean PI | 4.965 | 0.174 | 3 |
| Abundance | Type + Mean PIN | Type*Mean PIN | 5.444 | 0.142 | 3 |
| Abundance | Type + NPN | Type*NPN | 7.031 | 0.071 | 3 |
| Abundance | Type + NPNN | Type*NPNN | 6.190 | 0.103 | 3 |
| Richness | Type + Status | Type*Status | 8.157 | 0.227 | 6 |
| Richness | Type + Mean PI | Type*Mean PI | 2.092 | 0.554 | 3 |
| Richness | Type + Mean PIN | Type*Mean PIN | 2.832 | 0.418 | 3 |
| Richness | Type + NPN | Type*NPN | 5.561 | 0.135 | 3 |
| Richness | Type + NPNN | Type*NPNN | 2.638 | 0.451 | 3 |

PI = phylogenetic isolation. PIN = phylogenetic isolation from natives. NPN = nearest phylogenetic neighbour distance. NPNN = nearest phylogenetic native neighbour distance. See Methods for details on the different phylogenetic isolation indices.

**Supplementary Table 4:** Negative binomial models describing the effects of host plant native status, and/or host plant phylogenetic isolation, and several control variables (pollinator models only) on local-scale insect abundance.

| **Covariate/Contrast** | *z* | ***p*** |
| --- | --- | --- |
| **VORTIS SAMPLING [ALL PLANTS]** | | |
| Model 1: **Vortis Insect Abundance ~ Status**  [D² = 0.205, AIC = 409.4] | | |
| Status (Overall) | NA | NA |
| Status Contrasts: C-N | -1.342 | 0.372 |
| Status Contrasts: E-N | -3.520 | 0.001 |
| Status Contrasts: E-C | -2.092 | 0.092 |
| Model 2: **Vortis Insect Abundance ~ Status + Mean PI**  [D² = 0.209, AIC= 411.2] | | |
| Status (Overall) | NA | NA |
| Status Contrasts: C-N | -1.281 | 0.406 |
| Status Contrasts: E-N | -3.514 | 0.001 |
| Status Contrasts: E-C | -2.180 | 0.075 |
| Mean PI | 0.513 | 0.608 |
| Model 3: **Vortis Insect Abundance ~ NPN**  [D² = 0.108, AIC = 412.8] | | |
| NPN | -2.800 | 0.005 |
| **VORTIS SAMPLING [ NON-NATIVE PLANTS ONLY]** | | |
| Model 4: **Vortis Insect Abundance ~ Status + Mean PI from Natives**  [D² = 0.111, AIC = 261.2] | | |
| Status (E-C) | -1.794 | 0.073 |
| Mean PI from Natives | -0.148 | 0.883 |
| Model 5: **Vortis Insect Abundance ~ NPN from Natives**  [D² = 0.103, AIC = 259.4] | | |
| NPN from Natives | -1.93 | 0.054 |
| **POLLINATORS [ALL PLANTS]** | | |
| Model 6: **Pollinator Abundance ~ log(Replicates)**  [D² = 0.485, AIC = 636.4] | | |
| log(Replicates) | 10.886 | <1e-04 |
| Model 7: **Pollinator Abundance ~ log(Replicates) + Flowering Units + Status**  [D² = 0.624, 0.280 – 0.286, AIC = 620.43] | | |
| log(Replicates) | 10.859 | <1e-04 |
| Flowering Units | 5.350 | <1e-04 |
| Status Overall | NA | NA |
| Status Contrasts: C-N | 0.882 | 0.651 |
| Status Contrasts: E-N | -1.324 | 0.382 |
| Status Contrasts: E-C | -2.171 | 0.076 |
| Model 8: **Pollinator Abundance ~ log(Replicates) + Flowering Units + Mean PI**  [D² = 0.632, 0.209 – 0.381, AIC = 617.01] | | |
| log(Replicates) | 9.863 | <1e-04 |
| Flowering Units | 4.803 | <1e-04 |
| Mean PI | -3.156 | 0.002 |
| Model 9: **Pollinator Abundance ~ log(Replicates) + Flowering Units + NPN**  [D² = 0.611, 0.215 – 0.363, AIC = 620.79] | | |
| log(Replicates) | 9.863 | <1e-04 |
| Flowering Units | 5.275 | <1e-04 |
| NPN | -1.621 | 0.105 |
| **POLLINATORS [NON-NATIVE PLANTS ONLY]** | | |
| Model 10: **Pollinator Abundance ~ log(Replicates) + Flowering Units + Status + Mean PI from Natives**  [D² = 0.641, 0.275 – 0.429, AIC = 391.87] | | |
| log(Replicates) | 7.120 | <1e-04 |
| Status (E-C) | 1.918 | 0.055 |
| Flowering Units | 4.671 | <1e-04 |
| Mean PI from Natives | -1.668 | 0.095 |
| Model 11: **Pollinator Abundance ~ log(Replicates) + Flowering Units + NPN from Natives**  [D² = 0.595, 0.229 – 0.369, AIC = 395.22] | | |
| log(Replicates) | 7.552 | <1e-04 |
| Flowering Units | 4.764 | <1e-04 |
| NPN from Natives | -0.898 | 0.369 |

Contrasts calculated via post-hoc Tukey tests. N = native, C = congener, E = exotic, PI = phylogenetic isolation, NPN = nearest phylogenetic neighbour distance. D² represents the proportion of deviance explained by a model. D represents the range of deviance explained by all predictors of interest, after accounting for sampling effort (log(Replicates)) in pollinator models. Host plant native status did not significantly improve Model 8 (likelihood ratio test χ2 = 5.134, p = 0.077, d.f. = 2). See Methods for an explanation of the different phylogenetic isolation indices, of the calculation of D, and of the model building process.

**Supplementary Table 5:** Negative binomial models describing the effects of host plant native status, and/or host plant phylogenetic isolation, and several control variables (pollinator models only) on local-scale insect richness.

| **Covariate/Contrast** | ***z*** | ***p*** |
| --- | --- | --- |
| **VORTIS SAMPLING [ALL PLANTS]** | | |
| Model 1**: Vortis Insect Richness ~ Status**  [D² = 0.163, AIC = 259.6] | | |
| Status Overall | NA | NA |
| Status Contrasts: C-N | -1.915 | 0.134 |
| Status Contrasts: E-N | -2.841 | 0.013 |
| Status Contrasts: E-C | -0.843 | 0.676 |
| Model 2: **Vortis Insect Richness ~ Status + Mean PI**  [D² = 0.210, AIC = 259.0] | | |
| Status Overall | NA | NA |
| Status Contrasts: C-N | -1.943 | 0.127 |
| Status Contrasts: E-N | -3.172 | 0.004 |
| Status Contrasts: E-C | -1.187 | 0.461 |
| Mean PI | 1.622 | 0.104 |
| Model 3: **Vortis Insect Richness ~ NPN**  [D² = 0.112, AIC = 260.1] | | |
| NPN | -2.389 | 0.017 |
| **VORTIS SAMPLING [NON-NATIVE PLANTS ONLY]** | | |
| Model 4: **Vortis Insect Richness ~ Mean PI from Natives**  [D² = 0.008, AIC = 169.9] | | |
| Mean PI from Natives | 0.497 | 0.619 |
| Model 5: **Vortis Insect Richness ~ NPN from Natives**  [D² = 0.036, AIC = 169.1] | | |
| NPN from Natives | -1.075 | 0.282 |
| **POLLINATORS [ALL PLANTS]** | | |
| Model 6: **Pollinator Richness ~ log(Replicates)**  [D² = 0.560, AIC = 350.6] | | |
| log(Replicates) | 8.825 | <1e-04 |
| Model 7: **Pollinator Richness ~ log(Replicates) + Flowering Units + Status**  [D² = 0.636, D = 0.160 – 0.264, AIC = 342.6] | | |
| log(Replicates) | 8.938 | <1e-04 |
| Flowering Units | 2.506 | 0.012 |
| Status Overall | NA | NA |
| Status Contrasts: C-N | -0.534 | 0.854 |
| Status Contrasts: E-N | -2.966 | 0.008 |
| Status Contrasts: E-C | -2.411 | 0.042 |
| Model 8: **Pollinator Richness ~ log(Replicates) + Flowering Units + Status + Mean PI**  [D² = 0.671, D = 0.154 – 0.449, AIC = 337.4] | | |
| log(Replicates) | 8.236 | <1e-04 |
| Flowering Units | 2.059 | 0.039 |
| Status Overall | NA | NA |
| Status Contrasts: C-N | -0.746 | 0.736 |
| Status Contrasts: E-N | -2.781 | 0.015 |
| Status Contrasts: E-C | -2.030 | 0.105 |
| Mean PI | -2.579 | 0.010 |
| Model 9: **Pollinator Richness ~ log(Replicates) + Flowering Units + NPN**  [D² = 0.611, D = 0.120 – 0.389, AIC = 340.7] | | |
| log(Replicates) | 8.228 | <1e-04 |
| Flowering Units | 2.213 | 0.027 |
| NPN | -3.016 | 0.003 |
| **POLLINATORS [NON-NATIVE PLANTS ONLY]** | | |
| Model 10: **Pollinator Richness ~ log(Replicates) + Flowering Units + Status + Mean PI from Natives**  [D² = 0.665, D = 0.169 – 0.368, AIC = 207.8] | | |
| log(Replicates) | 7.025 | <1e-04 |
| Flowering Units | 2.296 | 0.022 |
| Status (E-C) | -2.326 | 0.020 |
| Mean PI from Natives | -1.414 | 0.157 |
| Model 11: **Pollinator Richness ~ log(Replicates) + Flowering Units + NPN from Natives**  [D² = 0.628, D = 0.121 – 0.311, AIC = 210.8] | | |
| log(Replicates) | 6.823 | <1e-04 |
| Flowering Units | 2.032 | 0.042 |
| NPN from Natives | -2.065 | 0.039 |

Contrasts calculated via post-hoc Tukey tests. N = native, C = congener, E = exotic, PI = phylogenetic isolation, NPN = nearest phylogenetic neighbour distance. D² represents the proportion of deviance explained by a model. D represents the range of deviance explained by all predictors of interest, after accounting for sampling effort (log(Replicates)) in pollinator models. Host plant native status did not significantly improve Model 4 (likelihood ratio test χ2 = 1.069, p = 0.301, d.f. = 1). See Methods for an explanation of the different phylogenetic isolation indices, of the calculation of D, and of the model building process.

**Supplementary Table 6:** Poisson/negative binomial models describing the effects of host plant native status, neophyte host plant arrival date, host plant phylogenetic isolation, and host plant range size (no. of hectads) on geographic-scale DBIF insect richness.

| **Covariate/Contrast** | ***z*** | ***p*** |
| --- | --- | --- |
| **ALL PLANTS [NEGATIVE BINOMIAL MODELS]** | | |
| Model 1: **DBIF Insect Richness ~ log(Sources)**  [D² = 0.920, AIC = 4512.1] | | |
| log(Sources) | 69.623 | <1e-04 |
| Model 2: **DBIF Insect Richness ~ log(Sources) + Hectads + Status**  [D² = 0.924, D = 0.007 – 0.830, AIC = 4485.4] | | |
| log(Sources) | 58.739 | <1e-04 |
| No. of Hectads | 4.881 | <1e-04 |
| Status Overall | NA | NA |
| N - ARCH | -0.263 | 0.962 |
| NEO - ARCH | -1.373 | 0.350 |
| NEO - N | -1.492 | 0.290 |
| Model 3: **DBIF Insect Richness ~ log(Sources) + Hectads + Mean PI**  [D² = 0.924, D = 0.009 – 0.828, AIC = 4484.3] | | |
| log(Sources) | 58.895 | <1e-04 |
| No. of Hectads | 5.539 | <1e-04 |
| Mean PI | -1.254 | 0.210 |
| Model 4: **DBIF Insect Richness ~ log(Sources) + Hectads + NPN**  [D² = 0.924, D = 0.010 – 0.829, AIC = 4476.8] | | |
| log(Sources) | 59.115 | <1e-04 |
| No. of Hectads | 5.386 | <1e-04 |
| NPN | -3.057 | 0.002 |
| **NON-NATIVE PLANTS ONLY [POISSON MODELS]** | | |
| Model 5: **DBIF Insect Richness ~ log(Sources)**  [D² = 0.919, AIC = 1316.7] | | |
| log(Sources) | 53.794 | <1e-04 |
| Model 6: **DBIF Insect Richness ~ log(Sources) + Hectads + Status**  [D² = 0.927, D = 0.025 – 0.763, AIC = 1293.5] | | |
| log(Sources) | 46.383 | <1e-04 |
| No. of Hectads | 3.091 | 0.002 |
| Status (NEO – ARCH) | -4.142 | <1e-04 |
| Model 7: **DBIF Insect Richness ~ log(Sources) + Hectads + Mean PI from Natives**  [D² = 0.924, D = 0.014 – 0.769, AIC = 1301.4] | | |
| log(Sources) | 46.731 | <1e-04 |
| No. of Hectads | 2.697 | 0.007 |
| Mean PI from Natives | -2.989 | 0.003 |
| Model 8: **DBIF Insect Richness ~ log(Sources) + Hectads + Mean PI from Natives + Status**  [D² = 0.927, D = 0.016 – 0.795, AIC = 1293.6] | | |
| log(Sources) | 44.701 | <1e-04 |
| No. of Hectads | 2.854 | 0.004 |
| Mean PI from Natives | -1.360 | 0.174 |
| Status (NEO – ARCH) | -3.109 | 0.002 |
| Model 9: **DBIF Insect Richness ~ log(Sources) + Hectads + NPN from Natives**  [D² = 0.924, D = 0.016 – 0.758, AIC = 1301.0] | | |
| log(Sources) | 46.961 | <1e-04 |
| No. of Hectads | 2.899 | 0.004 |
| NPN from Natives | -3.034 | 0.002 |
| Model 10: **DBIF Insect Richness ~ log(Sources) + Hectads + NPN from Natives + Status**  [D² = 0.927, D = 0.017 – 0.784, AIC = 1293.6] | | |
| log(Sources) | 45.109 | < 2e-16 |
| No. of Hectads | 2.966 | 0.003 |
| NPN from Natives | -1.370 | 0.171 |
| Status (NEO – ARCH) | -3.063 | 0.002 |
| **NEOPHYTE PLANTS ONLY [POISSON MODELS]** | | |
| Model 11: **DBIF Insect Richness ~ log(Sources)**  [D² = 0.914, AIC = 775.2] | | |
| log(Sources) | 37.830 | <1e-04 |
| Model 12: **DBIF Insect Richness ~ log(Sources) + Hectads + Time Since Neophyte Arrival**  [D² = 0.923, D = 0.012 – 0.850, AIC = 767.7] | | |
| log(Sources) | 25.438 | <1e-04 |
| No. of Hectads | 2.939 | 0.003 |
| Neophyte Arrival Date | 0.948 | 0.343 |

Contrasts calculated via post-hoc Tukey tests. N = native, C = congener, E = exotic, PI = phylogenetic isolation, NPN = nearest phylogenetic neighbour distance. D² represents the proportion of deviance explained by a model. D represents the range of deviance explained by all predictors of interest, after accounting for sampling effort (log(Sources)). Host plant native status did not significantly improve Models 3 and 4 (Model 3 likelihood ratio test χ2 = 1.737, p = 0.420, d.f. = 2; Model 4 likelihood ratio test χ2 = 0.853, p = 0.653, d.f. = 2). See Methods for an explanation of the different phylogenetic isolation indices, of the calculation of D, and of the model building process.

**Supplementary Table 7:** Beta models describing the effects of host plant native status, and/or host plant phylogenetic isolation, and pollinator host plant replicate number, on local-scale insect community distinctiveness.

| **Covariate/Contrast** | ***z*** | ***p*** |
| --- | --- | --- |
| **VORTIS SAMPLING [ALL PLANTS]** | | |
| Model 1: **Vortis Insect Community Distinctiveness ~ Status**  [Psuedo R² = 0.182, AIC = -63.3] | | |
| Status Overall | NA | NA |
| Status Contrasts: C-N | 2.586 | 0.026 |
| Status Contrasts: E-N | 2.833 | 0.013 |
| Status Contrasts: E-C | 0.162 | 0.986 |
| Model 2: **Vortis Insect Community Distinctiveness ~ Status + Mean PI**  [Psuedo R² = 0.187, AIC = -61.5] | | |
| Status Overall | NA | NA |
| Status Contrasts: C-N | 2.590 | 0.026 |
| Status Contrasts: E-N | 2.676 | 0.020 |
| Status Contrasts: E-C | 0.055 | 0.998 |
| Mean PI | 0.521 | 0.602 |
| Model 3: **Vortis Insect Community Distinctiveness ~ NPN**  [Psuedo R² = 0.005, AIC = -56.4] | | |
| NPN | 0.465 | 0.642 |
| **POLLINATORS [ALL PLANTS]** | | |
| Model 4: **Pollinator Community Distinctiveness ~ log(Replicates)**  [Psuedo R² = 0.234, AIC = -91.0] | | |
| log(Replicates) | -3.797 | 1.46e-04 |
| Model 5: **Pollinator Community Distinctiveness ~ log(Replicates) + Flowering Units + Status \| Status + Median Julian Sampling Date**  [Psuedo R² = 0.235, AIC = -107.3] | | |
| log(Replicates) | -4.459 | <1e-04 |
| Flowering Units | -2.483 | 0.013 |
| Status Overall | NA | NA |
| Status Contrasts: C-N | 3.905 | 2.85e-04 |
| Status Contrasts: E-N | 4.055 | 1.53e-04 |
| Status Contrasts: E-C | 1.333 | 0.372 |
| Model 6: **Pollinator Community Distinctiveness ~ log(Replicates) + Flowering Units + Mean PI**  [Psuedo R² = 0.291, AIC = -92.3] | | |
| log(Replicates) | -4.132 | <1e-04 |
| Flowering Units | -1.826 | 0.068 |
| Mean PI | 0.973 | 0.331 |
| Model 7: **Pollinator Community Distinctiveness ~ log(Replicates) + Flowering Units + NPN**  **\| Median Julian Sampling Date**  [Psuedo R² = 0.291, AIC = -94.2] | | |
| log(Replicates) | -3.914 | <1e-04 |
| Flowering Units | -1.799 | 0.072 |
| NPN | 1.309 | 0.191 |

Contrasts calculated via post-hoc Tukey tests. N = native, C = congener, E = exotic, PI = phylogenetic isolation, NPN = nearest phylogenetic neighbour distance. D² represents the proportion of deviance explained by a model. Beta regression mean submodel test values are reported above. See Methods for an explanation of the different phylogenetic isolation indices and of the model building process, and for the distinction between beta regression mean and precision submodels.

The following χ2 statistics report the results of likelihood ratio tests used in the model building process. Host plant median volume did not significantly improve the mean submodel of Model 1 (χ2 = 0.042, p = 0.837, d.f. = 1) or the precision submodel (χ2 = 1.672, p = 0.196, d.f. = 1) and so was excluded from this and subsequent Vortis models. Host plant branching architecture did not significantly improve the mean submodel of Model 1 (χ2 = 0.274, p = 0.601, d.f. = 1) or the precision submodel (χ2 = 2.241, p = 0.134, d.f. = 1) and so was excluded from this and subsequent Vortis models. Host plant status did not significantly improve the precision submodel of Model 1 (χ2 = 2.800, p = 0.247, d.f. = 1), Model 2 (χ2 = 2.731, p = 0.255, d.f. = 1), or Model 6 (χ2 = 3.448, p = 0.178, d.f. = 1). Host plant status did not significantly improve the mean submodel of Model 6 (χ2 = 0.644, p = 0.725, d.f. = 1). Phylogenetic isolation did not significantly improve the precision submodel of Model 2 (χ2 = 2.149, p = 0.143, d.f. = 1) or Model 6 (χ2 = 1.015, p = 0.314, d.f. = 1). Nearest phylogenetic neighbour distance did not significantly improve the precision submodel of Model 3 (χ2 = 0.148, p = 0.700, d.f. = 1) or Model 7 (χ2 = 1.833, p = 0.176, d.f. = 1). Median Julian sampling date did not significantly improve the mean submodel of Model 5 (χ2 = 0.086, p = 0.769, d.f. = 1), Model 6 (χ2 = 0.089, p = 0.766, d.f. = 1), or Model 7 (χ2 = 0.050, p = 0.823, d.f. = 1). Median Julian sampling date did not significantly improve the precision submodel of Model 6 (χ2 = 3.220, p = 0.073, d.f. = 1). Host plant flowering units did not significantly improve the precision submodel of Model 5 (χ2 = 0.143, p = 0.705, d.f. = 1), Model 6 (χ2 = 0.077, p = 0.782, d.f. = 1), or Model 7 (χ2 = 0.036, p = 0.851, d.f. = 1). log(replicates) did not significantly improve the precision submodel of Model 4 (χ2 = 0.874, p = 0.350, d.f. = 1), Model 5 (χ2 = 0.236, p = 0.627, d.f. = 1), Model 6 (χ2 = 0.115, p = 0.734, d.f. = 1), or Model 7 (χ2 = 0.787, p = 0.375, d.f. = 1).

**Supplementary Table 8:** Beta models describing the effects of host plant phylogenetic isolation on geographic-scale DBIF insect community distinctiveness on non-natives plants.

| **Covariate/Contrast** | ***z*** | ***p*** |
| --- | --- | --- |
| **NON-NATIVE PLANTS ONLY** | | |
| Model 1: **DBIF Community Distinctiveness ~ Sources**  [Psuedo R² = 0.069, AIC = -201.4] | | |
| Sources | -1.919 | 0.055 |
| Model 2: **DBIF Community Distinctiveness ~ Sources + Status**  [Psuedo R² = 0.104, AIC = -201.5] | | |
| Sources | -2.135 | 0.033 |
| Status (NEO – ARCH) | 1.438 | 0.150 |
| Model 3: **DBIF Community Distinctiveness ~ Sources + Mean PI from Natives \| Mean PI from Natives**  [Psuedo R² = 0.277, AIC = -220.0] | | |
| Sources | -3.018 | 0.003 |
| Mean PI from Natives | 7.513 | <1e-04 |
| Model 4: **DBIF Community Distinctiveness ~ Sources + NPN from Natives \| NPN from Natives**  [Psuedo R² = 0.218, AIC = -211.1] | | |
| Sources | -2.674 | 0.008 |
| NPN from Natives | 5.050 | <1e-04 |
| **NEOPHYTE PLANTS ONLY** | | |
| Model 5: **DBIF Community Distinctiveness ~ Sources**  [Psuedo R² = 0.013, AIC = -99.6] | | |
| Sources | -0.450 | 0.653 |
| Model 6: **DBIF Community Distinctiveness ~ Time Since Neophyte Arrival**  [Psuedo R² = 0.010, AIC = -106.3] | | |
| Time Since Neophyte Arrival | 0.447 | 0.655 |

Contrasts calculated via post-hoc Tukey tests. NEO = neophyte, ARCH = archaeophyte, PI = phylogenetic isolation, NPN = nearest phylogenetic neighbour distance. Psuedo R² represents the proportion of variance explained by a model. Beta regression mean submodel test values are reported above. See Methods for an explanation of the different phylogenetic isolation indices and of the model building process, and for the distinction between beta regression mean and precision submodels.

The following χ2 statistics report the results of likelihood ratio tests used in the model building process. Host plant native status did not significantly improve the mean submodel of Model 1 (χ2 = 2.028, p = 0.154, d.f. = 1), Model 3 (χ2 = 0.006, p = 0.939, d.f. = 1), or Model 4 (χ2 = 0.034, p = 0.854, d.f. = 1). Host plant native status did not significantly improve the precision submodel of Model 1 (χ2 = 0.599, p = 0.439, d.f. = 1), Model 2 (χ2 = 0.022, p = 0.883, d.f. = 1), Model 3 (χ2 = 0.077, p = 0.781, d.f. = 1), or Model 4 (χ2 = 0.070, p = 0.791, d.f. = 1). Host plant range size did not significantly improve the mean submodel of Model 1 (χ2 = 1.121, p = 0.290, d.f. = 1) or the precision submodel (χ2 = 0.018, p = 0.892, d.f. = 1) and so was excluded from this and subsequent models. Sources did not significantly improve the mean submodel of Model 6 (χ2 = 1.177, p = 0.278, d.f. = 1), or the precision submodel of Model 1 (χ2 = 0.594, p = 0.441, d.f. = 1), Model 2 (χ2 = 0.022, p = 0.883, d.f. = 1), Model 3 (χ2 = 0.004, p = 0.949, d.f. = 1), Model 4 (χ2 = 0.029, p = 0.865, d.f. = 1), Model 5 (χ2 = 1.421, p = 0.233, d.f. = 1), or Model 6 (χ2 = 2.422, p = 0.120, d.f. = 1). Time since neophyte arrival did not significantly improve the precision submodel of Model 1 (χ2 = 0.917, p = 0.338, d.f. = 1) or Model 6 (χ2 = 1.238, p = 0.266, d.f. = 1).

**References**

Ahlmann-Eltze, C. (2017). ggsignif: Significance Brackets for 'ggplot2'. R package version 0.4.0.

Baines, C. (2000) How to Make a Wildlife Garden, 2^nd^ ed. Francis Lincoln, London.

Barbosa A. M., Brown J. A., Jimenez-Valverde A. & Real R. (2016). modEvA: Model Evaluation and Analysis. R package version 1.3.2.

Barnard, P. C. (2011) The Royal Entomological Society Book of British Insects. Royal Entomological Society, St Albans.

Buck, F. D. (1954) Coleoptera (Lagriidae, Alleculidae, Tetratomidae, Meladryidae, Salplingidae, Pythidae, Mycteridae, Oedemeridae, Mordellidae, Scraptiidae, Pyrochroidae, Rhipiphoridae, Anthicidae, Aderidae and Meloidae). Handbooks for the Identification of British Insects. Volume 5, Part 9. Royal Entomological Society, London.

Cribari-Neto, F. & Zeileis, A. (2010). Beta Regression in R. Journal of Statistical Software, 34(2),

1-24. URL http://www.jstatsoft.org/v34/i02/.

Dormann, C. F., Gruber B. & Fruend, J. (2008). Introducing the bipartite Package: Analysing Ecological Networks. *R News, 8/2*, 8-11.

Dowle, M. & Srinivasan, A. (2017) data.table: Extension of `data.frame`. R package version 1.10.4-3.

Duffy, E. A. J. (1953) Coleoptera (Scolytidae and Platypodidae). Handbooks for the Identification of British Insects. Volume 5, Part 15. Royal Entomological Society, London.

Fox, J. & Weisberg, S. (2019). An {R} Companion to Applied Regression, Third Edition. Thousand Oaks, USA: Sage.

Hackston, M. (2018) *Mike’s Insect Keys*. https://sites.google.com/site/mikesinsectkeys/Home [Accessed December 2017]

Hincks, W. D. (1949) Dermaptera and Orthoptera. Handbooks for the Identification of British Insects. Volume 1, Part 5. Royal Entomological Society, London.

Hodkinson, I. D. & White, I. M. (1979) Homoptera Psylloidea. Handbooks for the Identification of British Insects. Volume 11, Part 5a. Royal Entomological Society, London.

Hothorn, T., Bretz, F. & Westfall, P. (2008). Simultaneous Inference in General Parametric Models. *Biometrics, 50*(3), 346-363. doi:10.1002/bimj.200810425.

Hsieh, T. C., Ma, K. H. & Chao, A. (2016) iNEXT: An R package for interpolation and extrapolation of species diversity (Hill numbers). *Methods in Ecology and Evolution,* *7*(12), 1451-1456. doi:10.1111/2041-210X.12613

Hubble, D. (2012) Keys to the Adults of Seed and Leaf Beetles of the British Isles (Coleoptera: Bruchidae, Orsodacnidae, Megalopodidae & Chrysomelidae). FSC, Telford.

Joy, N. H. (1932) A Practical Handbook of British Beetles. 2 Vols. HF & G Witherby, London.

Kirk-Spriggs, A. H. (1996) Pollen Beetles. Coleoptera: Kateretidea and Nitidulidae: Meligethinae. Handbooks for the Identification of British Insects. Volume 5, Part 6a. Royal Entomological Society, London.

Le Quesne, W. J. (1965) Hemiptera Cicadomorpha Deltocephalinae. Handbooks for the Identification of British Insects. Volume 2. Part 2b. Royal Entomological Society, London.

Le Quesne, W. J. & Payne, K. R. (1981) Cicadellidae (Typhlocybinae) with a Checklist of the British Auchenorhyncha (Hemiptera, Homoptera) Handbooks for the Identification of British Insects. Volume 2, Part 2c. Royal Entomological Society, London.

Levey, B. (2009) British Scraptiidae. Handbooks for the Identification of British Insects. Volume 5, Part 18. Royal Entomological Society, St Albans.

Lüdecke, D. (2019). sjPlot: Data Visualization for Statistics in Social Science. doi: 10.5281/zenodo.1308157. R package version 2.6.3.

Luff, M. L. (2007) The Carabidae (Ground Beetles) of Britain and Ireland. Handbooks for the Identification of British Insects Volume 4, Part 2, 2^nd^ ed. Royal Entomological Society, St Albans.

Marshall, J. A. & Haes, E. C. M. (1988) Grasshoppers and Allied Insects of Great Britain and Ireland. Harley Books, Colchester.

Majerus, M. & Kearns, P. (1989) Ladybirds. Naturalists’ Handbooks 10. Richmond Publishing Co. Ltd., Slough.

Melo, A., S. (2017). CommEcol: Community Ecology Analyses. R package version 1.6.5.

Morris, M. G. (1990) Orthocerus Weevils. Coleoptera Curculionoidea (Nemonychidae, Anthribidae, Urodontidae, Attelabidae and Apionidae. Handbooks for the Identification of British Insects Volume 5, Part 16. Royal Entomological Society of London, London.

Morris, M. G. (1997) Broad-Nosed Weevils. Coleoptera: Curculionidae (Entiminae). Handbooks for the Identification of British Insects. Volume 5, Part 17a. Royal Entomological Society, London.

Morris, M. G. (2008) True Weevils (Part II). Coleoptera: Curculionidae, Ceutorhynchidae. Handbooks for the Identification of British Insects Volume 5, Part 17c. Royal Entomological Society, St Albans.

Oksanen, J., Blanchet, F. G., Friendly, M., Kindt, R., Legendre, P., McGlinn, D., … Wagner, H. (2018). vegan: Community Ecology Package. R package version 2.4-6.

Ritchie, M. E., Phipson, B., Wu, D., Hu, Y., Law, C.W., Shi, W., and Smyth, G. K. (2015). limma powers differential expression analyses for RNA-sequencing and microarray studies. *Nucleic Acids Research, 43*(7), e47. doi: 10.1093/nar/gkv007

Salisbury, A., Armitage, J., Bostock, H., Perry, J., Tatchell, M., & Thompson, K. (2015). Enhancing gardens as habitats for flower-visiting aerial insects (pollinators): Should we plant native or exotic species? *Journal of Applied Ecology, 52*(5), 1156-1164. doi:10.1111/1365-2664.12499

Salisbury, A., Al-Beidh, S., Armitage, J., Bird, S., Bostock, H., Platoni, A., … Perry, J. Enhancing gardens as habitats for plant-associated invertebrates: should we plant native or exotic species? *Biodiversity and Conservation,* *26*(11), 2657-2673. doi:10.1007/s10531-017-1377-x

Schneider, C. A., Rasband, W. S., & Eliceiri, K. W. (2012), "NIH Image to ImageJ: 25 years of image analysis", *Nature Methods, 9*, 671-675.

Southwood, T. R. E. & Leston, D. (1959) Land and Water Bugs of the British Isles . Frederick Warne, London.

Unwin, D. M. (1984) A Key to the Families of British Beetles. FSC Publications, Dorchester.

Unwin, D. M. (2001) A Key to the Families of British Bugs. FSC Publications, Dorchester

Venables, W. N. & Ripley, B. D. (2002) Modern Applied Statistics with S. Fourth Edition. Springer, New York. ISBN 0-387-95457-0

Wickham, H. (2007) Reshaping data with the reshape package. *Journal of Statistical Software, 21*(12).

Wickham, H. (2009) ggplot2: Elegant Graphics for Data Analysis. Springer-Verlag, New York.

Wickham, H. (2011) The Split-Apply-Combine Strategy for Data Analysis. *Journal of Statistical Software, 40*, 1-29.

Wickham, H. (2017). stringr: Simple, Consistent Wrappers for Common String Operations. R package version 1.2.0.

Zeileis, A. & Hothorn, T. (2002). Diagnostic Checking in Regression Relationships. *R News, 2*, 7-10.
